# Tissue-specific microRNA expression alters cancer susceptibility conferred by a *TP53* noncoding variant

**DOI:** 10.1038/s41467-019-13002-x

**Published:** 2019-11-07

**Authors:** Qipan Deng, Hui Hu, Xinfang Yu, Shuanglin Liu, Lei Wang, Weiqun Chen, Chi Zhang, Zhaoyang Zeng, Ya Cao, Zijun Y. Xu-Monette, Ling Li, Mingzhi Zhang, Steven Rosenfeld, Shideng Bao, Eric Hsi, Ken H. Young, Zhongxin Lu, Yong Li

**Affiliations:** 1 Department of Medicine, Section of Epidemiology and Population Sciences, Baylor College of Medicine, Houston, TX USA; 2 Department of Cancer Biology, Lerner Research Institute, Cleveland Clinic, Cleveland, OH USA; 3 Department of Medical Laboratory, Central Hospital of Wuhan, Wuhan, China; 4 Key Laboratory of Carcinogenesis and Invasion, Ministry of Education, Xiangya Hospital; Cancer Research Institute, Xiangya School of Medicine, Central South University; Key Laboratory of Carcinogenesis, Chinese Ministry of Health, Changsha, China; 5 Department of Pathology, Division of Hematopathology, Duke University Medical Center, Durham, NC USA; 6 Department of Oncology, the First Affiliated Hospital of Zhengzhou University; Lymphoma Diagnosis and Treatment Center of Henan Province, Zhengzhou, China; 7 Robert J. Tomsich Pathology and Laboratory Medicine Institute, Cleveland Clinic, Cleveland, OH USA

**Keywords:** Cancer genetics, Mutation

## Abstract

A noncoding polymorphism (rs78378222) in *TP53*, carried by scores of millions of people, was previously associated with moderate risk of brain tumors and other neoplasms. We find a positive association between this variant and soft tissue sarcoma. In sharp contrast, it is protective against breast cancer. We generated a mouse line carrying this variant and found that it accelerates spontaneous tumorigenesis and glioma development, but strikingly, delays mammary tumorigenesis. The variant creates a miR-382-5p targeting site and compromises a miR-325-3p site. Their differential expression results in p53 downregulation in the brain, but p53 upregulation in the mammary gland of polymorphic mice compared to that of wild-type littermates. Thus, this variant is at odds with Li-Fraumeni Syndrome mutants in breast cancer predisposition yet consistent in glioma predisposition. Our findings elucidate an underlying mechanism of cancer susceptibility that is conferred by genetic variation and yet altered by microRNA expression.

## Abstract (web summary)

TP53 mutations can cause increased risk for cancers. Here, the authors show a noncoding polymorphism in TP53 increases risk of some cancers but delays onset of others, and in a mouse model show this is via alteration of microRNA targeting sites that differ in impact depending on the tissue.

## Introduction

Li-Fraumeni syndrome (LFS; also called sarcoma, breast, leukemia, and adrenal gland [SBLA] syndrome in the past) is a rare, inherited familial predisposition to a wide range of cancers^1–4^. Mutations in the coding sequence (CDS) of *TP53*, encoding the p53 tumor suppressor, are found in ~75% of LFS families; these *TP53* mutants produce mutant p53 proteins that lack most or all tumor-suppressive functions and often confer oncogenic properties^5–7^. Changes in *TP53* noncoding sequences, in contrast, have lower penetrance but still confer cancer susceptibility. A noncoding single-nucleotide polymorphism (SNP, rs78378222) in *TP53* is associated with moderate risk of several cancers^8^. Located in the fifth nucleotide of the *TP53* polyadenylation signal (PAS), the minor allele of this SNP is C, resulting in an alternative PAS (AATACA) instead of the canonical PAS (AATAAA). Unlike LFS mutant and common *TP53* CDS variants, such as P72R and P47S^9,10^, this *TP53* noncoding variant produces wild-type (WT) p53 proteins, albeit at a lower level in cells^11^. Cancer susceptibility conferred by this *TP53* noncoding variant^8,12–15^ does not strictly mirror that of *TP53* germline coding mutations in LFS patients^3,16^ (Supplementary Table 1): individuals with the minor allele are at increased risk of brain tumors^8^ (particularly glioma^8,12–14,17^), neuroblastoma^18^, skin basal cell carcinoma (BCC)^8^, esophageal squamous cell carcinoma (SCC)^19^, prostate cancer, colorectal adenoma^8^, and uterine leiomyoma^20^.

In LFS patients, the risk of developing any invasive cancer (excluding skin cancer) is ~50% by age 30 (compared with 1% in the general population), and ~90% by age 70^21^. Although numerous tumor types are seen in patients with LFS, five core cancers (breast cancer, soft-tissue sarcoma, osteosarcoma, brain tumor, and adrenocortical carcinoma) make up ~80% of LFS-associated tumors^1–3^. Brain tumors occur in 9–16% of LFS patients, with glioma being the most common (>40%)^22,23^. Sporadically, glioma accounts for ~80% of all primary adult malignant brain tumors. That the risk of glioma is increased twofold in relatives of glioma patients provides evidence for an inherited risk^24^. A number of rare inherited cancer predisposition disorders, such as LFS, Turcot syndrome, and neurofibromatosis, are recognized to be associated with increased risk of glioma. Scores of common SNPs were recently identified as increasing the risk of glioma^15,17,25^. Supported by five independent studies^8,12–15,17^, the *TP53* noncoding variant (rs78378222[C]) increases glioma risk more significantly than for other tumors with an odds ratio (OR) ranging from 2.35 to 3.74 (Supplementary Table 1). It is estimated that this variant alone could represent up to 6% of the familial risk of glioma^13^. Thus, this *TP53* variant shares a phenotypic similarity with LFS mutants in brain tumor (glioma) predisposition.

Breast cancer is the most frequently reported tumor in adult LFS patients. Nearly 80% of LFS females develop breast cancer, whereas no LFS male does^22^. Even when male and female patients are considered together, breast cancer is found in 39% of patients, while soft-tissue sarcoma (the second most common tumor type) is found in 27% of patients^22^. This *TP53* noncoding variant does neither appear to increase the risk for sporadic breast cancer nor for high-risk breast cancer (Supplementary Table 1);^8^ however, in this study, all breast cancer patients and the vast majority of the unaffected controls were genotyped for this variant by imputation^8^, which has relatively low accuracy for infrequent alleles like this *TP53* variant^26^. In fact, for a variant with a frequency <2%, there is no such imputation method that achieves ≥95% concordance with Taqman real-time PCR or DNA sequencing^26–28^. In addition, other cancers to which patients with this variant are predisposed (neuroblastoma, prostate cancer, skin BCC, and esophageal SCC) occur infrequently in patients with LFS^3,29,30^.

In this study, we have performed direct genotyping of this *TP53* variant in patients with breast cancer and sarcoma. We uncover that this *TP53* variant increases the risk for soft-tissue sarcoma, but decreases the risk for breast cancer. We generate a mouse line carrying this variant and evaluate tumorigenesis in different organs; particularly, we investigate whether and how this variant increases the risk for glioma but not breast cancer. We have found that this *TP53* variant creates a targeting site for miR-382-5p (miR-382) that is highly expressed in the brain and compromises the site for miR-325-3p (miR-325) that is highly expressed in the mammary gland. Differential expression of these two microRNAs (miRNAs) is likely responsible for observed p53 upregulation in the mammary gland, but p53 downregulation in the brain of polymorphic mice as compared with wild-type littermates. Our findings uncover a *TP53* variant at odds with LFS mutants in regard to breast cancer risk yet consistent with LFS mutants in predisposition to glioma and reveal an underlying mechanism of tissue-specific cancer susceptibility that is mediated by miRNAs.

## Results

### Susceptibility to breast cancer and sarcoma

To determine the association of this *TP53* noncoding variant with the risk for breast cancer and sarcoma, we genotyped rs78378222 in adult patients with breast cancer (*n* = 2373) or sarcoma (*n* = 130) and their respective unaffected controls (*n* = 9972 and 8947) of Chinese Han descent (Table 1). The minor allele frequencies (MAFs) in the controls were 0.017–0.018, comparable with previous reports (Supplementary Table 1). We observed a significant association between rs78378222[C] and the risk of sarcoma (OR = 3.29, *P* = 0.0014, Cochran–Armitage trend test). The risk was limited to soft-tissue sarcoma (OR = 4.55, *P* = 3.3 × 10^−5^, Cochran–Armitage trend test), because no patients with osteosarcoma carried this variant. In sharp contrast, this *TP53* variant protected against breast cancer (OR = 0.573, *P* = 0.0078, Cochran–Armitage trend test).

**Table 1:** Association between breast cancer and sarcoma and rs78378222[C]

Cancer type	OR	*P*	95% CI	Case number	Frequency in cases (%)	Control number	Frequency in controls (%)
Breast cancer	0.573	0.0078	0.374–0.867	2373	0.0107	9972	0.0180
Sarcoma	3.29	0.0014	1.51–7.17	130	0.0538	8947	0.0170
Soft-tissue sarcoma	4.55	3.3 × 10^−5^	2.07–9.99	96	0.0729	8947	0.0170
Osteosarcoma	NA	NA	NA	34	0	NA	NA

*OR* odds ratio, *CI* confidence interval, *NA* not applicable

Controls were patients who were not diagnosed with any cancer

### A mouse model for this variant and spontaneous tumorigenesis

**Fig. 1:**
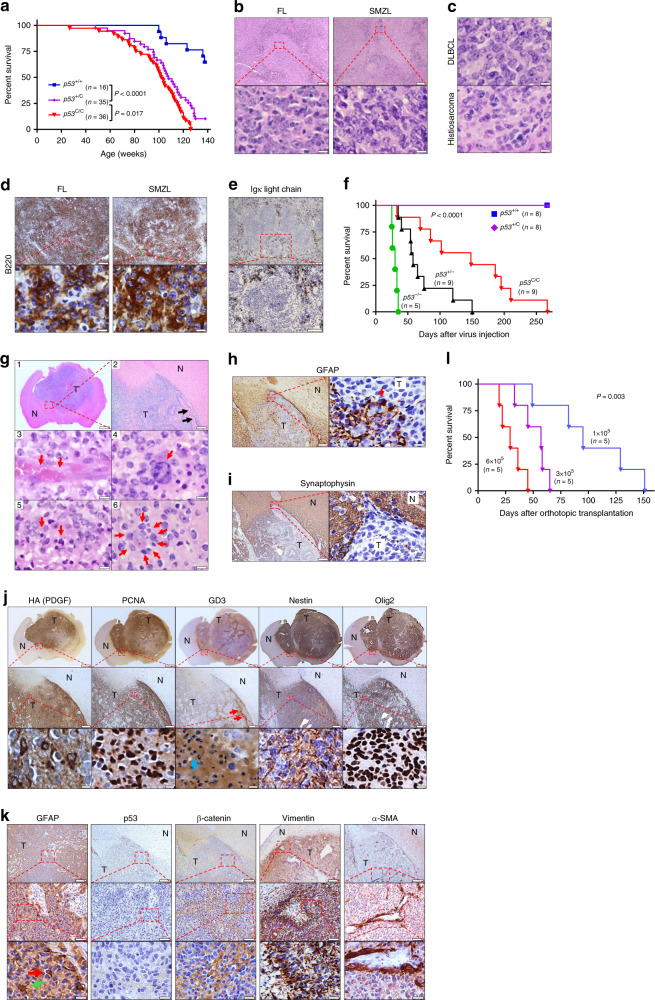
*p53*^*C*^ accelerates spontaneous tumorigenesis and gliomagenesis in mice. **a** Survival of p53 polymorphic mice. *P* < 0.0001 (log-rank test). **b** Hematoxylin and eosin stain (H&E) staining of representative follicular lymphoma (FL) and splenic marginal zone lymphoma (SMZL) in polymorphic mice. Scale bar = 200 μm (top), 20 μm (bottom). **c** Diffuse large B-cell lymphoma (DLBCL, top) and histiosarcoma (bottom). Scale bar = 20 μm. **d** Immunohistochemistry (IHC) staining for B-cell marker B220 in FL and SMZL. Scale bar = 200 μm (top), 20 μm (bottom). **e** IHC staining for Ig κ light chain of SMZL. Scale bar = 200 μm (top), 50 μm (bottom). **f** Survival of polymorphic mice injected in the right subventricular zone (SVZ) with retrovirus carrying *Pdgfb*. *P* < 0.0001 (log-rank). **g** H&E of glioma in *p53*^C/C^ mice (*n* = 9). **1**, a representative tumor (magnification 2 × ). Scale bar = 1 mm. **2**, invasive growth of the tumor cells. Arrows showing invasion front of the tumor. Scale bar = 100 μm. **3**, a blood vessel (arrows) in the tumor tissue (×1000). **4**, infiltrating monocytes (arrow) within tumors. **5**, Necrotic tumor cells (Arrows). **6**, highly invasive tumor cell (arrows) growth surrounding a neuron (star). Scale bar = 5 μm in 3–6. **h** IHC for the astrocyte/glial cell marker GFAP. Arrow showing a tumor cell staining positive for GFAP, but most tumor cells lost GFAP expression. **i** IHC of synaptophysin. Scale bar in (**h**, **i**) = 100 μm (left), 10 μm (right). **j** IHC of glioma stem cell markers in *p53*^C/C^ mice. *Pdgfb* was tagged with 3× hemagglutinin (HA). PCNA: tumor cell proliferation, GD3, Nestin, and Oligo2: neuralgia progenitor/glioma stem cell maker (red arrows point to the accumulation of GD3 at invasive front line; blue arrow: necrotic cells). Scale bars = 1 mm (top), 100 μm (middle), and 5 μm (bottom row). **k** IHC of other markers in *p53*^C/C^ mice. Green arrow: GFAP-positive cell with typical astrocyte morphology, red arrow: GFAP-positive tumor cell. Scale bars = 100 μm (top), 20 μm (middle), and 5 μm (bottom). N normal tissues, T tumors. Images were representative of staining six tumors (one per animal) (**g**–**k**). **l** Survival of syngeneic WT mice orthotopically transplanted with *p53*^C/C^ glioma cells (**f**). *P* = 0.003 (log-rank)

To investigate the causative nature of this variant in cancer, we generated a mouse line with a *Trp53* gene carrying the alternative PAS (Supplementary Fig. 1a, b). We termed the resultant polymorphic *Trp53* allele *Trp53*^1755C^ (abbreviated as *p53*^C^), because the 1755th nucleotide of mouse *p53* reference mRNA is the ortholog of human rs78378222. The animals were in the C57BL/6 genetic background. Mice carrying the *p53*^C^ allele had shorter survival than their WT *p53*^+/+^ littermates (Fig. 1a), with median survival of 108 weeks for heterozygous (*p53*^+/C^) and 102 weeks for homozygous (*p53*^C/C^) mice, but longer survival than *p53*^+/−^ mice (a median survival of ~70 weeks)^31^. We noted that *p53*^+/C^ and *p53*^C/C^ mice had close survival curves, yet *p53*^+/C^ mice survived better than *p53*^C/C^ mice (*P* = 0.017, log-rank test, Fig. 1a). Tumors developed more frequently in polymorphic mice than in WT controls (Supplementary Fig. 1c). B-cell malignancies were the most frequently occurring cancer in *p53*^+/C^ and *p53*^C/C^ mice (without development of thymic lymphoma) (Fig. 1b–e and Supplementary Table 2). Exome sequencing of lymphomas developed in polymorphic mice showed *p53* coding somatic mutations in all tumors, with a relative read ratio of *p53* and *Gapdh* close to 1:1 (Supplementary Table 3, Supplementary Fig. 1d). There were a few *p53* mutations in Supplementary Table 3 resembling human hotspot *TP53* pathogenic mutations. While we did not test these mutants using functional assays, most of them are likely pathogenic and others may be variants of uncertain significance. First, there were two point-nonsense mutants that will result in truncated and nonfunctional p53 proteins lacking DNA-binding domain, tetramerization domains, and extreme carboxyl terminus^32^. Second, P38T, the only recurrent mutation, is found in two tumors. This P residue is located in a proline-rich domain that is critical to the p53 transcriptional activity and is conserved in human, mouse, rat, dog, macaque, and chicken^33^. Third, four other mutants occur in the DNA-binding domain of the p53 protein that probably disrupt p53–DNA interaction^32^. Irradiation further shortened the survival of *p53* polymorphic mice, with increased cancer-associated death and increased thymic lymphoma incidence compared with untreated mice (Supplementary Fig. 1e–h). The median survival were 104, 93, and 85 weeks for irradiated *p53*^+/+^, *p53*^+/C^, and *p53*^C/C^ mice, respectively. These data indicate that this p53 noncoding variant increases the risk of malignancies and shortens survival.

### Glioma

*TP53* is the only gene that (1) causes a rare monogenic Mendelian disorder (LFS) that includes a greatly increased risk of glioma, and (2) has a common inherited SNP (rs78378222) associated with a smaller increased risk of glioma (Supplementary Table 1). Glioblastoma (GBM) is the highest grade astrocytoma. This *TP53* variant predispose both GBM and non-GBM glioma^17^. Approximately 30% of GBM tumors demonstrate a proneural gene expression pattern, characterized by frequent *TP53* mutation and *PDGFRA* mutation and/or overexpression^34^. We employed a PDGF-driven GBM mouse model with a proneural gene expression pattern by injecting a retrovirus expressing *Pdgfb* into the brain subventricular zone (SVZ)^35^. *p53*^−/−^ mice receiving retrovirus had the earliest tumor onset, and all succumbed to glioma within 40 days, whereas *p53*^+/−^ mice survived up to 150 days (Fig. 1f). All *p53*^C/C^ mice succumbed to glioma between 45 and 260 days, but neither *p53*^+/C^ nor *p53*^+/+^ mice with viral *Pdgfb* had developed any tumors by day 275 when they were all killed, and none showed any brain lesions. Tumors from *p53*^C/C^ mice were highly infiltrative and invasive with extensive vascularization and necrosis (Fig. 1g), all histological hallmarks of GBM. These tumors extensively expressed markers for GBM stem cells and the epithelial–mesenchymal transition (EMT) (Fig. 1h-k). The malignant phenotype of tumor cells was further demonstrated by transplanting them into syngeneic immunocompetent recipient mice in a dose-dependent manner (Fig. 1l). These data support that the *TP53* variant is pathogenic in gliomagenesis.

### Breast cancer

**Fig. 2:**
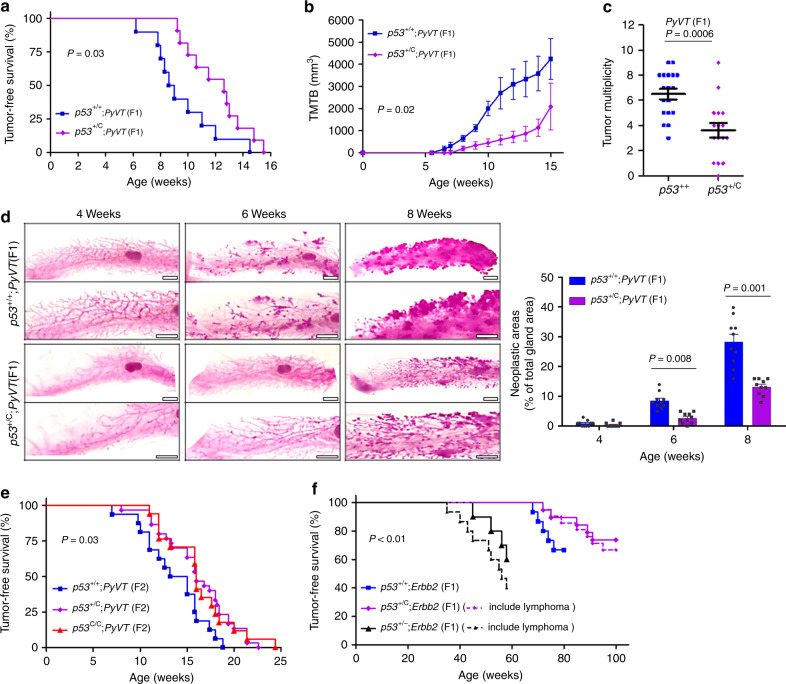
*p53*^C^ delays either *PyVT*- or *Errb2*-driven mammary tumorigenesis in mice. **a** Tumor-free survival of F1 female offspring of *p53*^+/C^ mice crossed with *PyVT*-overexpressing mice (FVB/N-Tg(MMTV-PyVT)634Mul/J). *n* = 10 for *p53*^+/+^;*PyVT* and *n* = 11 for *p53*^+/C^;*PyVT*; *P* *=* 0.03 (log-rank test). **b** Tumor volume in F1 female *p53*^+/+^;*PyVT* and *p53*^+/C^;*PyVT* mice at indicated ages. TMTB total measured tumor burden. *P* *=* 0.02 (Mann–Whitney *U* test). **c** Tumor multiplicity in F1 females at 15 weeks. The data were presented as mean ± s.e.m., error bars depict s.e.m.; *P* = 0.0006 (Mann–Whitney *U* test). **d** Whole-mount carmine red stain of the mammary glands from F1 females at indicated ages. *n* = 5 mice per group. Scale bar, 2 mm. On the right was the quantification of neoplastic/hyperplastic areas as a percentage of the total gland areas as shown (left) using image analysis. The data were presented as mean + s.e.m., error bars depict s.e.m. (*n* = 10 mammary gland from five mice per group) and analyzed with two-sided Student’s *t* test. **e** Tumor-free survival of F2 female offspring (obtained by crossing F1 *p53*^+/C^;*PyVT* with F1 *p53*^+/C^ mice). *P* *=* 0.03 (log-rank test). **f** Mammary tumor-free survival and tumor-free survival of F1 female offspring from *Erbb2* mice interbred with *p53*^+/C^ mice. As *p53*^+/C^ and p53^+/−^ mice may develop lymphoma, dashed lines indicated tumor-free survival (*P* = 0.006, log-rank test) and solid lines indicated mammary tumor-free survival (*P* *<* 0.001, log-rank test), i.e., dashed lines included 2 of the 21 *p53*^+/C^;*Erbb2* mice and 5 of the 15 p53^+/−^;*Erbb2* mice that developed lymphomas. *p53*^+/−^;*Erbb2* mice, as a control, were obtained by breeding *p53*^−/−^ mice with *Erbb2* mice

To determine the role of this *TP53* variant in the pathogenesis of breast cancer, we first crossed *p53*^+/C^ mice with mice expressing the polyoma virus middle T antigen (*PyVT*) driven by the mouse mammary tumor virus long terminal repeat (MMTV) promoter in the mammary gland^36^. *PyVT* mice were in the FVB background. We examined mammary tumorigenesis in the first filial generation (F1) hybrid mice (50:50 C57BL/6 to FVB background), and the F2 offspring produced by mating F1 mice to *p53*^+/C^ mice (75:25 C57BL/6 to FVB background, Supplementary Table 4). To our surprise, *p53*^+/C^;*PyVT* F1 females developed smaller and fewer mammary tumors and survived longer than *p53*^+/+^;*PyVT* F1 littermates (Fig. 2a–c). We examined the precancerous transformation of mammary epithelial cells using whole mounts of mammary gland and found that the *p53*^C^ allele delays development of epithelial hyperplasia to about 8 weeks of age, compared with 4 to 6 weeks in *p53*^+/+^;*PyVT* littermates (Fig. 2d). In F2 offspring, both *p53*^C/C^;*PyVT* and *p53*^+/C^;*PyVT* mice had longer tumor-free survival than *p53*^+/+^;*PyVT* littermates (Fig. 2e). Tumors from polymorphic mice were more differentiated and showed less EMT compared with tumors from *p53*^+/+^;*PyVT* mice at the same age, as evidenced by the higher expression of β-casein and E-cadherin (Supplementary Fig. 2a).

As most breast tumors that develop in LFS females are ERBB2-positive^22,37^, we next crossed *p53*^+/C^ mice with mice overexpressing MMTV-driven *Erbb2* in the FVB background and examined mammary tumorigenesis in F1 hybrid mice (50:50 C57BL/6 to FVB background). Similarly, *p53*^+/C^;*Erbb2* mice had longer tumor-free survival than the *p53*^+/+^;*Erbb2* littermates, whereas *p53*^+/−^;*Erbb2* mice had the shortest tumor-free survival (Fig. 2f). Tumors from *p53*^+/C^;*Erbb2* mice expressed higher levels of E-cadherin and pan cytokeratin (PCK) than the *p53*^+/+^;*Erbb2* control, indicating their epithelial origin and slower cancer progression in *p53* polymorphic mice (Supplementary Fig. 2b, c). Fewer *p53*^+/C^;*Erbb2* mice developed lung metastasis than *p53*^+/+^; *Erbb2* mice. There were fewer and smaller microscopic metastatic tumors in the lung per *p53*^+/C^;*Erbb2* animal than that per *p53*^+/+^;*Erbb2* animal (Supplementary Fig. 2d–g). p53 protein staining was strong in mammary tumors that developed in mice with either the polymorphic or WT *p53* (Supplementary Fig. 3). DNA sequencing of the *p53* gene in five mammary tumors from each group revealed all samples carried heterozygous *p53* coding mutations, supporting that gain-of-function p53 mutation is needed for mammary tumorigenesis.

### p53 deregulation and differential miRNA expression

**Fig. 3:**
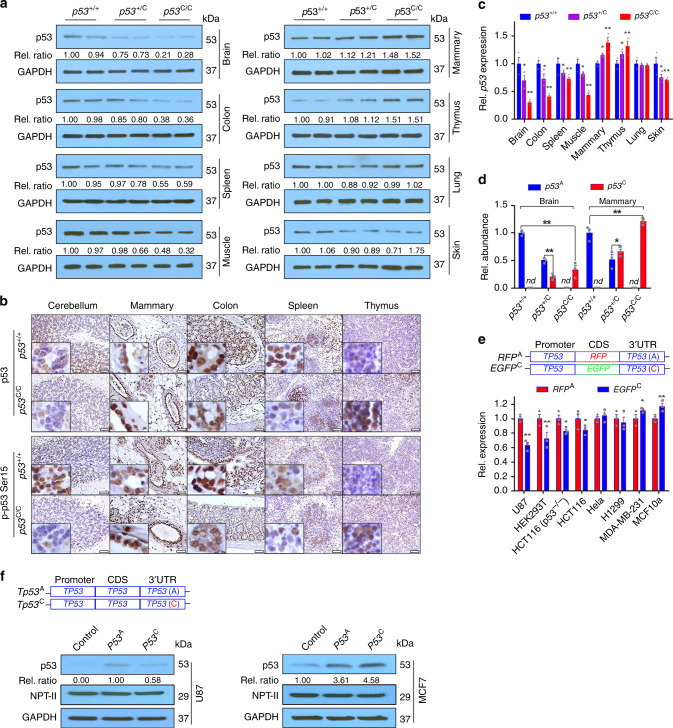
Differential p53 deregulation by the *p53*^C^ allele in tissues and cell lines. **a** Western blotting analyses for p53 protein levels in tissues from mouse littermates. Each pair of lanes indicates samples from two different mice. Rel. ratio: the relative signal densities of p53 as referenced to that of GAPDH. **b** IHC staining for p53 (upper panel) and phosphorylated p53 at site Ser15 (lower panel) in *p53*^+/+^ and *p53*^C/C^ mice. Mice were treated with 4 Gy total body γ-irradiation, and the brain, mammary gland, colon, spleen, and thymus were collected 6 h later for IHC staining. Scale bar, 50 μm. *n* = 3 mice per group, inserts show high-magnification (×400) images. **c** Quantitative real-time PCR (qPCR) analysis for *p53* mRNA levels in mouse tissues. Rel. *p53* expression: Relative *p53* mRNA levels normalized to *Gapdh*. The data were presented as mean ± s.e.m.; *n* = 3 independent biological replications. **d** Relative abundance (Rel. abundance) of A and C p53 cDNA alleles as determined by sequencing the PCR product of p53 cDNAs reverse transcribed from total mRNAs extracted from the brain and mammary tissues of mouse littermates. *nd* not detectable. **e** qPCR analysis for *EGFP/RFP* mRNA levels from indicated cell lines transfected with *EGFP*^C^ or *RFP*^A^, with *neomycin phosphotransferase* (NPT-II) as a reference, a gene carried by the vector. CDS, coding sequence. Rel. expression: Relative expression. The data were presented as mean ± s.e.m.; *n* = 3 biological triplicates for each cell line. **c**–**e** *P < 0.05, **P < 0.01 (one-way ANOVA). Error bars depict s.e.m. **f** Western blotting analyses for p53 protein levels in MCF7 and U87 cells transfected with a plasmid expressing *TP53* with a WT 3′UTR (*TP53*^A^) or a 3′UTR with an alternative PAS (*TP53*^C^). Rel. ratio: the relative signal densities of p53 as referenced to that of NPT-II. *n* = 3 biological triplicates for each cell line. **a**, **f** Source data from Western blotting are provided in a Source Data file

We analyzed p53 expression and activation in various tissues. p53 protein and mRNA levels, as well as protein phosphorylation upon irradiation, in the brain, colon, and spleen of *p53*^C/C^ mice were lower than in WT littermates (Fig. 3a–c). p53 protein in the brain of *p53*^C/C^ mice was significantly lower than that in *p53*^+/+^ mice (Fig. 3a), in line with observed gliomagenesis (Fig. 1f). In contrast, p53 was upregulated in the mammary gland of *p53*^C/C^ mice compared with WT littermates. When cDNA was prepared from the total RNAs in mouse tissues and sequenced, the *p53*^C^ alleles from *p53*^C/C^ mice were outnumbered by *p53*^A^ alleles from *p53*^+/+^ mice in the brain, whereas the *p53*^C^ allele outnumbered *p53*^A^ in the mammary gland (Fig. 3d). We cloned the whole native human *TP53* 3′UTR (the A allele, *TP53*^A^) or the 3′UTR with the alternative PAS (the C allele, *TP53*^C^) downstream to a EGFP or RFP (*EGFP*^C^ and *RFP*^A^) driven by the *TP53* promoter. These two constructs were introduced into cells, and the expression of EGFP and RFP was determined. The mRNA of *EGFP*^C^ were lower than that of *RFP*^A^ in U87 glioma cells, yet the opposite was observed in MCF10a and breast cancer MDA-MB-231 cells (Fig. 3e). When *EGFP/RFP* was replaced by the *TP53* coding sequence, the A allele resulted higher p53 expression than the C allele in U87, but not in MCF7 cells (Fig. 3f). Thus, the variant resulted in p53 upregulation or downregulation, dependent on the tissue or cellular context.

**Fig. 4:**
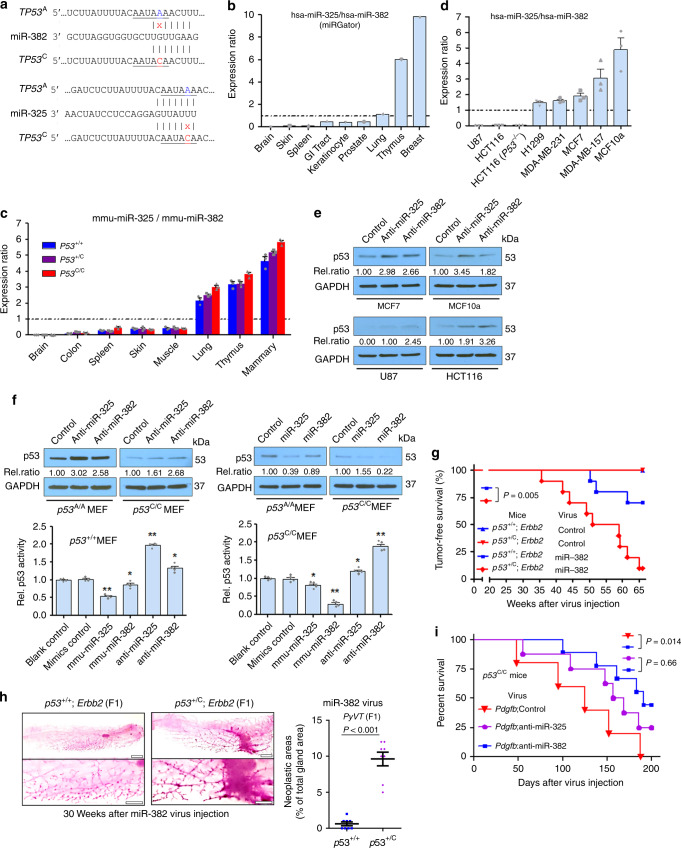
Tissue-specific miRNA expression results in differential *p53* deregulation. **a** The A (*TP53*^A^) to C (in red, *TP53*^C^) variation creates a miR-382 (hsa-miR-382-5p) site and compromises the miR-325 (hsa-miR-325-3p) site. **b** miR-325:miR-382 expression ratio in different human tissues, calculated by dividing the mean miR-325 read count by the mean miR-382 read count, using deep-sequencing data obtained from miRGator. **c** miR-325:miR-382 expression ratio in different mouse tissues using qPCR. **d** miR-325:miR-382 expression ratio in the indicated human cancer cell lines using qPCR. The data in (**c**, **d**) were presented as mean ± s.e.m.; error bars depict s.e.m. *n* = 3 biological triplicates for each cell line with three technical triplicates. **e** Representative western blotting analysis for p53 in human cell lines. **f** p53 expression (top) and reporter activity (bottom) in *p53*^+/+^ and *p53*^C/C^ MEFs transfected with miR-325 or miR-382 inhibitors or mimics. Control denotes the negative control #1 for mirVana miRNA inhibitors or mimics; Rel. ratio: the relative signal density of p53 as referenced to GAPDH (values were the average of three independent experiments). *P < 0.05, **P < 0.01 (one-way ANOVA). Error bars depict s.e.m. **g** Percent survival of *p53*^+/+^;*Erbb2* and *p53*^+**/**C^;*Erbb2* F1 female offspring overexpressing miR-382 in the mammary gland. Twenty-week-old mice (*n* = 10 per group) were injected in the third mammary gland pairs with lentivirus. *P* = 0.005 (log-rank). **h** Whole-mount carmine red stain of the mammary glands from *p53*^+/+^;*Erbb2* and *p53*^+/C^;*Erbb2* F1 females injected with lentivirus expressing miR-382. *n* = 4 mice per group. On the right was the quantification of neoplastic/hyperplastic areas as a percentage of the total gland areas as shown (left) using image analysis. The data were presented as mean + s.e.m. Error bars depict s.e.m. (*n* = 8 mammary gland from four mice per group) and analyzed with two-sided Student’s *t* test. **i** Survival of *p53*^C/C^ m**i**ce overexpressing *Pdgfb* and an inhibitor to miR-382. Mice were injected in the right SVZ with retrovirus expressing both *Pdgfb* and anti-miR-382 (*n* = 9), anti-miR-325 (*n* = 8), or an inhibitor control (*n* = 5). *P* *=* 0.014 (log-rank test). **e**, **f** Source data from Western blotting are provided in a Source Data file

Because the variant exerts moderate regulation over p53, we suspected that miRNA expression is a determinant of differential p53 deregulation in polymorphic mice. miR-325 putatively targets the native *TP53* 3′UTR, whereas miR-382 targets the *TP53* 3′UTR with the PAS variant in both human and mouse (Fig. 4a; Supplementary Fig. 4a). miR-325 and miR-382 expression varies markedly across different human tissues with miR-325 highly expressed in the breast, and miR-382 highly expressed in the brain (Supplementary Fig. 4b–f). The ratio of miR-325:miR-382 is lowest in the brain, and highest in the breast/mammary gland in both humans (Fig. 4b) and mice (Fig. 4c). Like miR-325, three other miRNAs putatively targeting *TP53*^A^ are also highly expressed in human breast tissues compared with the brain (Supplementary Fig. 5). The *p53*^C^ allele itself did not alter the expression of miR-325 or miR-382 in the brain, mammary gland, and other tissues in mice (Supplementary Fig. 4g, h). We next examined the expression of these two miRNAs in several human cell lines: U87 glioma and HCT116 colon cancer cells had the lowest miR-325: miR-382 ratio, compared with MCF10a, MCF7, and two other breast cancer cell lines (Fig. 4d; Supplementary Fig. 4i, j). We then determined miRNA regulation of endogenous p53 in these cells. miR-382 inhibition resulted in higher p53 protein expression than did miR-325 inhibition in U87 and HCT116 cells, which had higher endogenous miR-382 levels, while opposite results were observed in MCF7 and MCF10a cells, which had higher endogenous miR-325 levels (Fig. 4e). Similarly, miR-325 inhibition resulted in greater p53 protein expression than did miR-382 inhibition in *p53*^+/+^ MEFs, while opposite results were observed in *p53*^C/C^ MEFs (Fig. 4f).

We introduced miRNA mimics into MEFs and found miR-325 downregulated p53 to a greater extent than did miR-382 in *p53*^+/+^ MEFs, whereas miR-382 downregulated p53 to a greater extent than did miR-325 in *p53*^C/C^ MEFs (Fig. 4f). In human cells with exogenous *TP53*^A^, miR-325 downregulated p53 protein levels more than miR-382; in contrast, in cells with *TP53*^C^, miR-382 downregulated p53 protein levels more than miR-325 (Supplementary Fig. 6a–d). In ensuing reporter assays, modulation of miR-382 expression had a greater impact on *TP53*^C^-driven reporter activity than did miR-325 modulation, whereas modulation of miR-325 expression had a greater impact on *TP53*^A^-driven reporter activity than did miR-382 modulation (Fig. 4f, Supplementary Fig. 6e–k). Taken together, these data suggest that miR-325 targets the A allele more effectively than does miR-382, whereas miR-382 targets the C allele more effectively than does miR-325.

To test the contribution of miRNA-targeting sites (i.e., the PAS plus its adjacent nucleotides), we cloned the *TP53* coding sequence driven by the human *TP53* promoter, upstream of the SV40 3′UTR with either the WT PAS (A) or the PAS variant (C) (Supplementary Fig. 7a, b), neither of which are targeted by miR-382. These two plasmids were co-transfected into five cell lines, along with a p53 activity reporter construct. Regardless of the endogenous levels of miR-382, all five cell lines with the WT PAS displayed higher p53 activity than those with the alternative PAS (Supplementary Fig. 7c). Next, we generated EGFP-expressing plasmids with the *TP53* 3′UTR carrying either the WT PAS (A) or the PAS variant (C). We mutated the four nucleotides immediately downstream of the PAS (M4) to eliminate the miR-382 targeting site (Supplementary Fig. 7d–g). In U87 cells with high endogenous miR-382 levels (Fig. 4d), the A allele resulted in higher EGFP expression than did the C allele in U87 cells. When miR-382: *TP53* 3′UTR (C) was disrupted, cells with the M4 C allele had higher EGFP expression than those with the M4 A allele (Supplementary Fig. 7h). These results support that the integrity of the miR-382 binding site is essential to miR-382-mediated suppression of *TP53*^C^.

To further test direct interaction between *TP53*^A^/*TP53*^C^ and miR-325/miR-382, we performed pull-down assays using H1299 cells transfected with biotinylated RNA fragments and streptavidin beads to co-purify their binding partners (Supplementary Fig. 8). First, biotinylated *TP53* 3′UTR fragments containing miRNA-binding sites were used as baits to co-purify potential binding miRNAs (Supplementary Fig. 8a). The fragment containing the native *TP53* PAS pulled down more miR-325 than miR-382, whereas the one containing the alternative PAS pulled down more miR-382 than miR-325; when the binding sites for miR-325, miR-382, or both were mutated, the respective fragment was unable to pull down miR-325, miR-382, or either of them (Supplementary Fig. 8b-d). Second, biotinylated miRNAs were used to co-purify exogenous 3′UTR in H1299 cells transfected with EGFP-expressing plasmids containing the *TP53* 3′UTR carrying either the WT PAS or the alternative PAS with a WT or mutant miR-382 targeting site (Supplementary Fig. 7d, f; Supplementary Fig. 8e). The four plasmids resulted in comparable 3′UTR levels (Supplementary Fig. 8f). Biotinylated miR-325 pulled down the WT 3′UTR more effectively than did miR-382, while miR-382 pulled down the 3′UTR with the alternative PAS more effectively than did miR-325; elimination of the miR-382-binding site completely prevented the 3′UTR from being co-purified with miR-382 (Supplementary Fig. 8g). These experiments provide added evidence to support direct binding between *TP53*^A^/*TP53*^C^ and miR-325/miR-382.

### *p53*^C^ suppression by miR-382 in vivo

We next altered miRNA expression in the mouse brain and mammary gland, and evaluated changes in tumorigenesis. We chose miR-382 because it is highly abundant than miR-325 (Supplementary Fig. 4), and has no orthologs like miR-325 (Supplementary Fig. 5a). First, we injected a retrovirus overexpressing miR-382 into the mammary gland of F1 littermates with either *p53*^+/+^*;Erbb2* or *p53*^+/C^*;Erbb2*. By 65 weeks, miR-382 significantly accelerated mammary tumor development in *p53*^+/C^;*Erbb2* mice, but had smaller impact on *p53*^+/+^;*Erbb2* mice (Fig. 4g). *p53*^+/+^*;Erbb2* or *p53*^+/C^*;Erbb2* mice receiving control virus did not develop tumors. With miR-382 overexpression in the mammary gland, *p53*^+/C^;*Erbb2* mice had shorter tumor-free survival than *p53*^+/+^;*Erbb2* mice (Fig. 4g), in marked contrast to their untreated counterparts (Fig. 2f). Before tumors were visible, miR-382 overexpression in the mammary gland of *p53*^+/C^;*Erbb2* mice promoted mammary epithelial outgrowth compared with that in *p53*^+/+^;*Erbb2* mice (Fig. 4h). Second, we injected a retrovirus overexpressing both *Pdgfb* and an inhibitor to miR-382 into the SVZ of *p53*^C/C^ mice. Those receiving both *Pdgfb* and the miR-382 inhibitor prolonged the survival of *p53*^C/C^ mice compared with those receiving *Pdgfb* and the control inhibitor (or the miR-325 inhibitor) (Fig. 4i). This is likely due to miR-382 inhibition resulting in p53 upregulation at the target site of *p53*^C/C^ mice. These data demonstrate that modulating the expression of *p53*^C^-targeting miR-382 alters tumor development both in the brain and in the mammary gland of *p53*^C^ polymorphic mice.

## Discussion

The architecture of inherited cancer susceptibility is a montage of predisposition alleles with different levels of risk and prevalence (i.e., allele frequency) in the general population^38^. At one end of the spectrum are very rare to rare high-penetrance mutants causing Mendelian diseases, all of which are located in the CDS of tumor suppressor genes (e.g., *TP53* germline mutations in LFS). Their MAFs are typically ≤0.01%, and odds ratios (ORs) for cancer risk are as high as 20. Virtually all familial cancer syndromes are caused by these germline coding mutations. At the other end are common low-penetrance susceptibility alleles with MAFs >5% and ORs of 1.1–2.0. The vast majority of hundreds if not thousands of these low-penetrance alleles are located in noncoding regions of the human genome, where our understanding of biological consequences and cancer causality is rudimentary^38,39^. In comparison with other GWAS polymorphisms, the *TP53* noncoding variant (rs78378222[C]) is unique in cancer susceptibility, tissue-specificity, and pathogenicity. First, it is associated with an increased risk of multiple types of tumors with a MAF of 0.1–1.92% and ORs ranging from 1.39 to 4.55 (Supplementary Table 1). Given the world population is estimated to reach 7.7 billion this year and assuming the MAF is 1.0 %, characterization of this variant will benefit ~150 million people worldwide at the risk of cancer. Second, it, as a *TP53* variant in a Chinese population, protects breast cancer, the most frequent LFS tumor type and increases the risk for soft-tissue sarcoma, the second most frequent LFS tumor type. The *p53*^C^ variant delays mammary tumorigenesis in two mouse models. Third, it is bona fide pathogenic in comparison with other reported germline variants potentially disrupting miRNA binding. There are multiple studies on polymorphisms disrupting miRNA coding sequences^40^ or polymorphisms on the 3′UTR miRNA-binding sites of cancer genes, like *BRCA1*^41^, *ESR1*^42^, and *KRAS*^43^. Among them, the SNP rs61764370 (also known as LCS6) in a let-7 miRNA complementary site within the 3′UTR of the *KRAS* oncogene^43^ is among the most studied. However, the clinical utility of the KRAS LCS6 variant has been significantly questioned since the initial publications showing it is associated with lung cancer^43,44^, breast cancer^45^, and ovarian cancer^44^. In the largest ever study with a total of 140,012 human subjects, the KRAS LCS6 variant did not increase the risk of ovarian cancer or breast cancer, regardless of the BRAC1/2 status; null results were also obtained for associations with overall survival for ovarian cancer, breast cancer, and all other previously reported associations for these cancers^46^. Other than this *TP53* variant, few polymorphisms in cancer genes that potentially interact with miRNA have been validated using animal models. Therefore, ascertaining the pathogenic role of this *TP53* variant in this study facilitates our understanding of low-penetrance noncoding cancer susceptibility loci with a low frequency.

We compare cancer patients carrying rs78378222[C] to LFS patients. The rs78378222[C] differs from LFS mutants in several aspects: mutation sites (over 100 sites vs. a single nucleotide), tumor spectrum (including breast cancer vs. protective against breast cancer), penetrance (high vs. moderate), and the size of the affected population (reported thousands vs. potentially as many as 150 millions) (Table 2). Another difference is the impact of sex on cancer penetrance: the lifetime cancer risk of LFS patients is estimated to be 73% in males and ~100% in females, with the high risk of breast cancer accounting for the difference^47^. The effect of sex on cancer penetrance may not exist or could even be reversed in patients carrying this variant, which confers risk for prostate cancer and protection for breast cancer. Finally, different miRNAs are involved in regulation of cancer susceptibility by LFS mutants and the noncoding variant. Relative hypomethylation of the promoter of *miR-34A*, a p53 target gene, is found in peripheral blood cells from LFS patients compared with that from patients with a wild-type *TP53*; hypermethylation of the *miR-34A* promoter is detected in tumors, but not in histologically normal adjacent tissues from LFS patients^48^. These results support of a role of miR-34a in inter-individual cancer susceptibility, yet the causative relationship has not been investigated^48^. This work, on the other hand, offers an example of miRNAs that are differentially expressed and modulate intra-individual tissue-specific cancer susceptibility. This study shows that differential expression of miRNAs reduces the expression of a pathogenic genetic variant in one tissue yet increases its expression in another and contributes, at least partially, to tissue-specific cancer susceptibility within the same individual. Collectively, these differences, particularly that these patients with the noncoding variant are protected from breast cancer, argue against calling rs78378222[C] an LFS variant. In the clinical context of cancer clusters found in a family that fit the description (i.e., multiple family members developed various cancers associated with this variant as in Supplementary Table 1), rs78378222 genotyping and a potential targeted cancer surveillance plan, different from the one for LFS^49^, should be considered for those at risk.

**Table 2:** *TP53* germline coding mutations and the noncoding variant in cancer patients

Characteristic	LFS^a^	PAS^b^
Altered loci	Coding (>100 mutation sites)	Noncoding (1 site)
Tumor spectrum	Breast cancer	Protective against breast cancer [h, m]
	Brain tumor (glioma)	Brain tumor (glioma) [h, m]
	Soft-tissue sarcoma	Soft-tissue sarcoma [h]
	Osteosarcoma	Prostate cancer [h]
	Adrenocortical carcinoma	Colorectal adenoma [h]
	Choroid plexus tumor	Skin basal cell carcinoma [h]
	Rhobdomyosarcoma of embryonal anaplastic subtype	Neuroblastoma [h]
		Esophageal squamous cell carcinoma [h]
		Uterine leiomyoma [h]
Mean age at tumor onset	~25 years	>>25 years
Population frequency	~0.01%	~2.0%
Sex effect on penetrance	Female > Male	Male > female?
Epigenetic regulation in susceptibility	miR-34a	miR-382 and miR-325

^a^The cancer spectrum in LFS was determined by comparing the incidence in LFS patients with that in the general population with similar demographics. Cancers like colorectal, prostate, and lung cancer and leukemia are found in LFS patients, yet they are not considered LFS-specific core cancer types because their incidence in LFS patients is not significantly higher than that in the general population. Li-Fraumeni-like (LFL) syndrome is similar to LFS, but does not meet the stringent classification criteria, i.e., the classic LFS definition established by Drs. Li and Fraumeni in 1988^4^. Birch^30, 65^ and Eeles^66^ have suggested two definitions of LFL, respectively. Regardless of the disease definition, *TP53* coding mutations are found in 20–40% of LFL families, and like LFS, breast cancer is the most frequent tumor type in LFL patients^67^. That patients with the *TP53* coding mutation have a later cancer onset than those with LFS mutations implies the influence of confounding genetic modifiers and the environment

^b^The cancer spectrum was determined by genome-wide association studies (GWAS) of cancer cases and unaffected controls (Supplementary Table 1). The late tumor onset is suggested by our association study and mouse model data. Specific to breast cancer, the variant increases the risk in a Chinese population, but has no effect in a European population. [h, m] denotes that the results are supported by both human and mouse data

A strength of our study is the use of direct genotyping in determining cancer susceptibility rather than genetic imputation. Another strength is that the biological plausibility of this variant in cancer risk and protection is extensively interrogated and ascertained using multiple mouse models and molecular cellular assays. However, our study has several limitations. First, the number of cases for soft-tissue sarcoma is relatively small. Second, there could be some species- or ethnic-specific differences in cancer predisposition. *p53*^+/C^ mice developed no glioma with *Pdgfb* overexpression in the SVZ. The *p53*^C^ allele delayed mammary tumorigenesis in two mouse models. We caution that although littermate mice were used, we cannot rule out the possibility that a gene or genes linked to the *p53*^C^ allele in the C57BL/6 strain influenced the decrease in mammary cancer risk. The variant is protective for breast cancer in this study with a Chinese population, yet there was no specific association between this variant and breast cancer in a European population^8^. This could be due to the inaccuracy of SNP imputation^8^ or ethnicity-specific differences in breast cancer protection. Third, there are many lingering questions to be addressed. What types of tumors do patients with *TP53*^C/C^ and children and adolescents with the variant develop? Are miRNAs behind the protective role of the *p53* variant against squamous cell carcinoma of head and neck^50^? Is p53 upregulated in some tissues to an extent so that carriers show moderate signs of ribosomopathies or the CHARGE syndrome, in which p53 is inappropriately activated^51,52^? Further studies from population genetics and mouse models will reveal and corroborate the broader disease spectrum associated with this noncoding variant that may affect up to 150 million people worldwide.

## Methods

### Data reporting

No statistical methods were used to predetermine sample size.

### Patients

All recruited breast cancer and sarcoma patients, who are ethnic Han Chinese, were diagnosed at the Central Hospital of Wuhan, China. Patients who were suspected to have LFS or hereditary breast cancer were excluded. Controls were unaffected (not diagnosed with any cancer) and were recruited at the same hospital with age (in 10-year intervals), sex, and ethnicity matched to the cancer cases. This study was approved by the Ethical and Scientific Committee of the Central Hospital of Wuhan, and all human subject research was performed in accordance with institutional, national, and Declaration of Helsinki requirements. This study was compliant with the “Guidance of the Ministry of Science and Technology for the Review and Approval of Human Genetic Resources”. There was no export of human genetic materials or data from China to the United States. The association of rs78378222[C] with the risk of cancer was determined using the Cochran–Armitage trend test.

### Genotyping

Peripheral blood samples were collected with informed consent, which was obtained from all study participants. Genotyping for rs78378222 was performed using the Taqman SNP Real-time PCR Assay (ThermoFisher Scientific, Waltham, MA). Less than 1% of the DNA samples failed the Taqman PCR genotyping, and were genotyped using Sanger DNA sequencing. We arbitrarily selected 100 samples from the Taqman genotyping and directly sequenced the allele—DNA sequencing results were in 100% concordance with the Taqman results.

### Reagents

Antibodies against B220 (ab64100, 1:50 dilution), Igκ light chain (ab190484, 1:100 dilution), GFAP (ab7260, 1:1000 dilution), synaptophysin (ab32127, 1:400 dilution), HA-tag (ab24779, 1:1000 dilution), PCNA (ab29, 1:10,000 dilution), GD3 (ab11779, 1:1000 dilution), nestin (ab6142, 1:1000 dilution), olig2 (ab109186, 1:100 dilution), E-cadherin (for IHC, ab76055, 1:200 dilution), PCK (ab7753, 1:250 dilution), p53 (for IHC, ab31333, 1:50 dilution), phosphorylated-p53 Ser15 (for IHC, ab1431, 1:50 dilution), vimentin (ab92547, 1:200 dilution), NTP-II (ab33595, 1:250 dilution) and α-SMA (ab5694, 1:50 dilution) were purchased from Abcam (Cambridge, MA). Antibodies against p53 (for western blotting [WB], CST2524, 1:1000 dilution), phosphorylated-p53 Ser15 (for WB, CST9248, 1:1000 dilution), GAPDH (CST5174, 1:1000 dilution), and β-actin (CST4970, 1:5000 dilution) were from Cell Signaling Technology (Danvers, MA). Antibodies against β-casein (SC-166684, 1:20 dilution) and β-catenin (SC-7963, 1:50 dilution) were from Santa Cruz Biotechnology (Dallas, TX). miRNA mimics and anti-miRNAs for miR-382, miR-325, and their controls (mirVana miRNA Mimic Negative Control #1 and Anti-miR Negative Control #1) were purchased from ThermoFisher Scientific (Waltham, MA). Lentiviral vectors expressing miRNA mimics and anti-miRNAs for miR-382, miR-325, and control miRNA were purchased from Sigma-Aldrich.

### Cell culture

Human glioblastoma multiforme cell line U87 (HTB-14, ATCC, Manassas, VA), colon cancer cell line HCT116 (ATCC CCL-247), HCT116 (p53^−/−^, courtesy of Dr. Bert Vogelstein), cervical cancer cell line HeLa (ATCC CCL-2), non-small cell lung cancer cell line H1299 (ATCC CRL-5803), breast cancer cell line MDA-MB-231 (ATCC CRM-HTB-26), MCF7 (ATCC HTB-22), embryonic kidney cell line HEK293(ATCC CRL-1573), and 293GP cells (Clontech) were cultured in high glucose (5 g per liter) Dulbecco’s modified Eagle’s medium (DMEM) and 10% FBS with penicillin and streptomycin at 37 °C in 5% CO_2_. Human breast epithelial cell line MCF10a (ATCC CRL-10317) was cultured in complete MEGM media (Lonza, Walkersville, MD) with 100 ng/ml cholera toxin at 37 °C in 5% CO_2_. All cell lines were authenticated by the vendors using short-tandem repeat profiling, tested *Mycoplasma* free by the vendors and Cleveland Clinic Cell Culture Core, and used for <10 passages upon acquisition. We performed SNP genotyping for all these human cell lines and found that none of them carried rs78378222[C]. All cell culture experiments were performed with approved protocols by the institution biosafety committee of Cleveland Clinic.

### Mice

All mice were housed in microisolator cages (maximum five per cage of same-sex animals) and maintained in climate/temperature- and photoperiod-controlled barrier rooms (22 ± 0.5 °C, 12–12 h dark–light cycle) with unrestricted access to water and standard rodent diet (Teklad 2918, Harlan, Indianapolis, IN) unless otherwise indicated. The number of animals used in each experiment was estimated from published studies with statistically significant results. All mouse studies were performed in compliance with all relevant ethical regulations for animal testing and research using procedures approved by the Institutional Animal Care and Use Committees at Cleveland Clinic and Central Hospital of Wuhan. The genetically modified PAS mutation knock-in mouse was generated by using zinc-finger nuclease technology (Fox Chase Cancer Center, Philadelphia, PA). The *p53*^1175A^ located in the *p53* PAS (AATAAA) was mutated to *p53*^1175C^ (AATACA), corresponding to human rs78378222. The targeting vector was electroporated into C57BL/6 embryonic stem cells and male chimaeras were bred with C57BL/6 WT females, and resulting F1 heterozygotes interbred to generate homozygotes in C57BL/6 background. The whole *p53* exons from the founders were sequenced in their entirety to confirm no mutations other than the targeted nucleotide. Genotyping was done using forward primer 5′-CTC CAG GGC CTA CTT TCC TT-3′ and reverse primer 5′-GGT AAG GAC CAT GTG CCA GT-3′, followed by ApoI and EcoRV endonuclease double digestion after PCR and was confirmed by Sanger DNA sequencing. WT (*p53*^+/+^), heterozygous PAS (*p53*^+/C^), and homozygous PAS (*p53*^C/C^) mice were generated exclusively by breeding heterozygotes (*p53*^+/C^) in the C57BL/6 background (mice were born at the expected Mendelian ratio). When heterozygous PAS (*p53*^+/C^) mice were bred to *PyVT* (FVB/N-Tg(MMTV-PyVT)634Mul/J, #002374, Jackson Laboratory) or *Erbb2* (FVB/N-Tg(MMTVneu)202Mul/J, #002376) in the FVB genetic background, F1 or F2 littermates were used for comparison and analyses (Supplementary Table 4). When p53^−/−^ and *p53*^+/−^ mice (B6.129S2-*Trp53*^*tm1Tyj*^/J) were used, they were either in the C57BL/6 background (Fig. 1f) or bred to a hybrid C57BL/6:FVB background (with *Erbb2* mice, Fig. 2f; Supplementary Table 4). We used both male and female mice in all experiments, except in mammary tumorigenesis studies where only females were used. For mammary tumorigenesis, mice were monitored weekly before palpable tumors developed and the tumor width (*W*) and length (*L*) were measured twice a week using a caliper ^53^. Single tumor volume was calculated using the formula *V* = (*W* × *W* × *L*)/2. TMTB (total measured tumor burden) was calculated by summing up the volumes of all tumors developed in each animal. For lung metastasis in *Erbb2* mice, the average size of nodules (total nodule area [mm^2^]/total nodule number) was determined and converted to the average diameter (mm)^54^.

### Histopathology and immunohistochemistry

For routine histological analysis, all studied tissues from mice were fixed in 10% neutral buffered formalin (Sigma-Aldrich), embedded in paraffin, and evaluated by conventional H&E staining of serial sections cut at 5-μm thickness for pathological analyses. For IHC staining, deparaffinized and rehydrated sections were boiled in Na-citrate buffer (10 mM, pH 6.0) for 20 min for antigen retrieval. Sections were incubated with primary antibodies overnight at 4 °C. Tissue sections were developed using the rabbit-specific HRP/DAB (ABC) Detection IHC Kit or Mouse on Mouse Polymer IHC Kit (Abcam). After haematoxylin counterstaining, the slides were dehydrated and mounted. For IHC, positive staining was indicated as a dark brown signal in the cells. Images were then acquired using an Olympus IX51 microscope and analyzed using cellSens Dimension software (Olympus, Center Valley, PA).

### Retrovirus production

293GP cells (Clontech, Mountain View, CA) were seeded to 70% confluence in the DMEM medium containing 10% fetal bovine serum (FBS). pQCXIX vector was used to express the PDGFB–IRES–EGFP^35^. This viral plasmid and a plasmid-expressing VSVG were mixed with lipofectamine 2000 (ThermoFisher) according to the manufacturer’s instructions, and were added to the cell culture. After 4–6 h incubation, the medium was changed using fresh DMEM medium containing 10% FBS and then collected 48 h later. After filtration, the medium was centrifuged at 30,000 *g* for 2.5 h at 4 °C. The pellets were re-suspended in serum-free DMEM, and aliquots were frozen and stored at −80 °C until use. The viral titer was then determined by infecting cells with serial dilutions of medium containing retrovirus in tenfold increments. EGFP-expressing cells were then counted 48 h later, and viral titer was determined by the lowest dilution that gave rise to EGFP-expressing cells.

### Intracerebral stereotaxic injection

The glioma model was generated by injecting retrovirus expressing *Pdgfb* to the mouse SVZ^35^. The mice were anesthetized with an intraperitoneal injection of ketamine/xylazine (100 mg per kg and 10 mg per kg respectively) and placed in a stereotaxic frame. An approximately 1 -cm incision was made in the midline of the scalp to expose the bregma, and stereotaxic coordinates were determined. A burr hole was drilled through the skull, and a Hamilton (Hamilton Company, Reno, NV) syringe containing the retrovirus was then inserted into the brain. For targeting the dorsal lateral corner of the SVZ, we used the coordinates of 1 mm + 1 mm + 2.1 mm^35^, with the bregma as the reference. A volume of 1 µl was injected at 0.2 µL per min. At the end of the injection, the needle was slowly retracted. The skin was sealed with Gluture Topical Tissue Adhesive (ThermoFisher). Following surgery, the mice were placed on a warming pad to recover. In the immediate post-op period, the animals were continuously monitored until fully awake. During the recovery period, animals were re-examined at 12 and 24 h post-op. After the first 24 h post-op, the observation schedule became once daily with weight, appearance, and behavior being monitored. For transplantation experiments, primary tumor cells from *p53*^C/C^ mice were prepared as reported^55^ and injected into the SVZ of WT syngeneic recipient C57Bl/6 mice at a flow rate of 0.2 µl per minute. To downregulate the expression of miR-382 in the brain of *p53*^C/C^ mice, retrovirus carrying both *Pdgfb*^35^ and the Sigma-Aldrich MISSION miRNA Inhibitor (anti-miR-382) were injected into the mouse SVZ. The control inhibitor was Sigma-Aldrich MISSION miRNA Inhibitor Negative Control #1 (anti-ath-miR416; ath-miR416 is a miRNA from *Arabidopsis thaliana* with no homology to human and mouse miRNA or other gene sequences).

### Intraductal injection of mammary gland

Intraductal injections were performed as followed procedure^56–58^. Briefly, mice were anesthetized using ketamine/xylazine (100 mg per kg and 10 mg per kg, respectively), and hair was removed in the nipple area with a commercial hair removal cream. Eighteen microliters of high-titer lentivirus expressing miR-382 mixed with 2 μL 0.2% Evans blue dye (Sigma-Aldrich) in PBS was injected in the third mammary gland using a 34-gauge needle (Hamilton). The titer of the viral particles expressing miR-382 was 9.1 × 10^8^ transducing units (TU) per mL with TUs measured by p24 antigen ELISA assays. For the control, the same amount (TUs) of control viral particles was used. Mice were handled in a biological safety cabinet under a stereoscope.

### Survival and tumor burden studies

In accordance with IACUC guidelines, animals were allowed to live until humane endpoints were reached. Signs of terminal brain, mammary, and other tumor burden included the following: weight loss of 20%; peri-orbital hemorrhages; papilledema; epistaxis (nose bleeds); seizures; decreased alertness; impaired motor function; and/or impaired ability to feed secondary to decreased motor function, paresis or coma; changes in posture or ambulation—tense, stiff gait, ataxia, avoidance/inability to bear weight for 48 h, difficulty walking, inability to maintain upright position; restlessness; pacing; lethargy; tumor necrosis; systemic infection; signs of moderate-to-severe pain or distress; severe anemia; respiratory distress; or severe bleeding.

### Whole mount of mouse mammary gland

For whole-mount analysis, the fourth inguinal mammary glands were used by dissecting the inguinal mammary gland and fixing it on a microscope slide. The gland was immediately placed on a slide with Carnoy’s solution; blunt tweezers were used to gently configure the gland to its native orientation. The gland was fixed overnight before being subjected to re-hydration and carmine red staining^59,60^. The stained glands were then flattened, dehydrated, cleared in xylene, and mounted for microscopy.

Quantification of the neoplastic/hyperplastic mammary lesion at each time point as a ratio of the neoplastic/hyperplastic area vs. the total glandular area was performed^61^ using the ImageJ software (National Institutes of Health, Bethesda, MD)^62^.

### Western blotting analysis

Cell and tissue lysates were denatured in Laemmli sample buffer (Bio-Rad, Hercules, CA) and resolved by Tris-glycine SDS-PAGE (4–20% polyacrylamide, Mini-PROTEAN Precast Gels, Bio-Rad). After transfer to the polyvinyl difluoride membrane, the membranes were probed with primary antibodies, followed by incubation with horseradish peroxidase-conjugated secondary antibodies for detection with Pierce ECL western blotting substrate (ThermoFisher). Immunoblots were performed at least three times, and representative blots reported. All intensity quantification for western blot was performed using ImageJ software (Dr. Wayne Rasband, NIH, Bethesda, MD). The relative ratio of p53 with GAPDH, β-actin, or NPT-II as a reference was determined by calculating the relative density of the p53 bands normalized to that of the references. The relative ratio indicates the mean values of three independent blots in human cell experiments. Uncropped and unprocessed scans of all blots are provided in a Source Data file.

### RNA preparation and qPCR

To measure gene expression, the total RNA was extracted from cultured cell lines or tissues and tumors dissected from mice using Trizol (Invitrogen) and run on a Synergy HTX Multi-Mode Microplate Reader (BioTek, Winooski, VT) to ensure that the A260/A280 ratio was in the range of 1.8–2.2 and the rRNA ratio (28 S/18 S) was >0.9. RNA was reverse transcribed with the iScript Reverse Transcription Supermix kit (Bio-Rad). Real-time PCR was performed using the iQ SYBR Green Supermix (Bio-Rad) and CFX96 Real-Time PCR detection system (Bio-Rad). The primers used for real-time PCR were designed based on the Universal Probe Library (Roche, Roche Life Science, Pleasanton, CA, USA) with GAPDH as a reference. Real-time detection of miRNA expression was performed by using MystiCq miRNA qPCR Assay Primer (Sigma-Aldrich), using Universal Mouse Reference RNA and U6 as reference for mouse and human cells, respectively.

### FACS analysis of apoptosis and EGFP/RFP expression

Cells were plated in six-well plates at a density of 2 × 10^5^ cells per well and cultured in the DMEM and 10% FBS with penicillin and streptomycin until 80% confluence before treatment. Once confluence was reached, cells were collected and incubated with Alexa Fluor 488 Annexin V and propidium iodide using the Alexa Fluor 488 Annexin V/Dead Cell Apoptosis Kit (ThermoFisher). Cells were analyzed on a flow cytometer (MACSQuant Analyzer 10, Miltenyi Biotec, Bergisch Gladbach, Germany), and data were processed with the MACSQuantify software (Miltenyi Biotec). To detect EGFP/RFP expression, cells were co-transfected with EGFP/RFP-expressing plasmids containing the WT or the alternative PAS *TP53* 3′UTR (Fig. 3), or co-transfected EGFP-expressing plasmids containing the WT or the alternative PAS *TP53* 3′UTR with a RFP-expressing plasmid with WT *SV40* 3′UTR as transfection control (Supplementary Fig. 7), using Lipofectamine LTX (ThermoFisher) according to the manufacturers’ protocols. The mean fluorescence intensity (MFI) of EGFP was normalized to that of RFP.

### miRNA targeting and luciferase assay

miRNA-targeting prediction was performed using TargetScanS^63^ and PolymiRTS^64^. To determine p53 activity, cells were co-transferred with pNL(NLucP/p53-RE/Hygro) (Promega, Madison, WI) and the desired p53-expressing plasmids. A luciferase assay was then performed using the Nano-Glo Luciferase Assay System (Promega). Samples were then run on Synergy HTX Multi-Mode Microplate Reader (BioTek) to record the signal intensity. This pNL(NLucP/p53-RE/Hygro) plasmid contains several copies of a p53 response element (p53-RE) that drives transcription of a destabilized form of NanoLuc luciferase, an engineered small (23.3 kDa) luciferase fusion protein. The NlucP reporter consists of NanoLuc luciferase with a C-terminal fusion to PEST, a protein destabilization domain, which responds more quickly and with greater magnitude to changes in transcriptional activity than unmodified NanoLuc luciferase.

### RNA pull-down assays

5′ end biotin-labeled *TP53* 3′UTR RNA fragments with different PAS and other mutations and miRNA of interest labeled with biotin at the 3′end were commercially synthesized (Sigma-Aldrich). H1299 cells were seeded 1 day before transfection in 10 -cm tissue culture dish at 50% confluent. Twenty four hours later, cells were transfect with 5′ end biotinylated *TP53* 3′UTR RNA fragments (final concentration of 100 nM) or 3′ end biotinylated miRNA (final concentration of 100 nM), and/or a plasmid carrying a *TP53* 3′UTR (10 µg) according to the manufacturer’s guideline. Twenty four hours post transfection, cells were lysed in 550 μl of lysis buffer supplemented with protease inhibitors and RNase inhibitor, followed by incubation on ice for 10 min. The cell lysates were centrifuged at 4 °C, 14,000 *g* for 10 min, and the supernatant was subjected to Pierce™ Streptavidin Magnetic Beads in the presence of 2% RNase-free BSA (ThermoFisher Scientific) and 2% yeast tRNA (ThermoFisher Scientific) to block nonspecific binding. After 2 h of incubation, the beads were placed on a magnetic stand for 8 min and washed four times with 1 ml of lysis buffer. Purified RNAs were extracted by RNAzol^®^ RT (RN 190, Molecular Research Center; Cincinnati, OH) and subjected to reverse transcription and quantitative real-time PCR to determine the beads-bound miRNAs or 3′UTRs.

### Statistical and data analysis

The product-limit method of Kaplan and Meier was used for generating mouse survival or tumor-free survival curves, which were compared by using the log-rank (Mantel–Cox) test. An unpaired two-tailed Student’s *t* test was performed for two-group comparisons. One-way analysis of variance (ANOVA) was performed for multiple group comparisons with one independent variable, and two-way ANOVA for multiple group comparisons with two independent variables (genotype or treatment). Tumor multiplicity was compared using the Mann–Whitney *U* test. A *P*-value <0.05 was considered statistically significant for all data sets. All statistical analyses were performed using SPSS 16 (IBM Analytics, Chicago, IL), GraphPad Prism 5 (GraphPad Software, La Jolla, CA), or Sigmastat 4 (Systat Software, London, UK).

### Reporting summary

Further information on research design is available in the Nature Research Reporting Summary linked to this article.

## Supplementary information

Supplementary Information

Reporting Summary

## Source Data

Source Data

## Data Availability

The *p53* mutation data in mice with spontaneous lymphomas have been deposited in the NCBI BioProject database under the accession code PRJNA575686. The source data underlying Figs. 3a, f, 4e, f, and Supplementary Fig 6a–d are provided in a Source Data file. The *p53* polymorphic mouse line is available from Y.L. to any researcher upon request. All the other data supporting the findings of this study are available within the article and its supplementary information files and from the corresponding authors upon reasonable request. A reporting summary for this article is available as a Supplementary Information file.

## References

[1] Li, F. P. & Fraumeni, J. J. F. Soft-tissue sarcomas, breast cancer, and other neoplasmsA familial syndrome? *Ann. Intern. Med.* **71**, 747–752 (1969). 5360287 10.7326/0003-4819-71-4-747

[2] Lalloo, F. et al. Prediction of pathogenic mutations in patients with early-onset breast cancer by family history. *Lancet* **361**, 1101–1102 (2003). 12672316 10.1016/S0140-6736(03)12856-5

[3] Gonzalez, K. D. et al. Beyond Li Fraumeni syndrome: clinical characteristics of families with p53 germline mutations. *J. Clin. Oncol.* **27**, 1250–1256 (2009). 19204208 10.1200/JCO.2008.16.6959

[4] Li, F. P. et al. A cancer family syndrome in twenty-four kindreds. *Cancer Res.* **48**, 5358–5362 (1988). 3409256

[5] Oren, M. & Rotter, V. Mutant p53 gain-of-function in cancer. *Cold Spring Harb. Perspect. Biol.* **2**, a001107 (2010). 20182618 10.1101/cshperspect.a001107 PMC2828285

[6] Vogelstein, B. & Kinzler, K. W. Cancer genes and the pathways they control. *Nat. Med*. **10**, 789–799 (2004). 15286780 10.1038/nm1087

[7] Petitjean, A. et al. Impact of mutant p53 functional properties on TP53 mutation patterns and tumor phenotype: lessons from recent developments in the IARC TP53 database. *Hum. Mutat.* **28**, 622–629 (2007). 17311302 10.1002/humu.20495

[8] Stacey, S. N. et al. A germline variant in the TP53 polyadenylation signal confers cancer susceptibility. *Nat. Genet.* **43**, 1098–1103 (2011). 21946351 10.1038/ng.926 PMC3263694

[9] Dumont, P., Leu, J. I., Della Pietra, A. C. 3rd, George, D. L. & Murphy, M. The codon 72 polymorphic variants of p53 have markedly different apoptotic potential. *Nat. Genet.* **33**, 357–365 (2003). 12567188 10.1038/ng1093

[10] Jennis, M. et al. An African-specific polymorphism in the TP53 gene impairs p53 tumor suppressor function in a mouse model. *Genes Dev.* **30**, 918–930 (2016). 27034505 10.1101/gad.275891.115 PMC4840298

[11] Li, Y. et al. Single nucleotide variation in the TP53 3’ untranslated region in diffuse large B-cell lymphoma treated with rituximab-CHOP: a report from the International DLBCL Rituximab-CHOP Consortium Program. *Blood* **121**, 4529–4540 (2013). 10.1182/blood-2012-12-471722 PMC3668486 23515929

[12] Egan, K. M. et al. Rare TP53 genetic variant associated with glioma risk and outcome. *J. Med. Genet.* **49**, 420–421 (2012). 22706378 10.1136/jmedgenet-2012-100941 PMC3576847

[13] Enciso-Mora, V. et al. Low penetrance susceptibility to glioma is caused by the TP53 variant rs78378222. *Br. J. Cancer* **108**, 2178–2185 (2013). 23571737 10.1038/bjc.2013.155 PMC3670481

[14] Wang, Z. et al. Further confirmation of germline glioma risk variant rs78378222 in TP53 and its implication in tumor tissues via integrative analysis of TCGA data. *Hum. Mutat.* **36**, 684–688 (2015). 25907361 10.1002/humu.22799 PMC4750473

[15] Rice, T. et al. Understanding inherited genetic risk of adult glioma - a review. *Neurooncol Pr.* **3**, 10–16 (2016). 10.1093/nop/npv026 PMC4774334 26941959

[16] Guha, T. & Malkin, D. Inherited TP53 mutations and the Li–Fraumeni syndrome. *Cold Spring Harb. Perspect. Med* **7**, a026187 (2017). 28270529 10.1101/cshperspect.a026187 PMC5378014

[17] Melin, B. S. et al. Genome-wide association study of glioma subtypes identifies specific differences in genetic susceptibility to glioblastoma and non-glioblastoma tumors. *Nat. Genet*. **49**, 789–794 (2017). 28346443 10.1038/ng.3823 PMC5558246

[18] Diskin, S. J. et al. Rare variants in TP53 and susceptibility to neuroblastoma. *J. Natl. Cancer Ins.* **106**, dju047 (2014). 10.1093/jnci/dju047 PMC3982892 24634504

[19] Zhou, L., Yuan, Q. & Yang, M. A functional germline variant in the P53 polyadenylation signal and risk of esophageal squamous cell carcinoma. *Gene* **506**, 295–297 (2012). 22800615 10.1016/j.gene.2012.07.007

[20] Rafnar, T. et al. Variants associating with uterine leiomyoma highlight genetic background shared by various cancers and hormone-related traits. *Nat. Commun.* **9**, 3636 (2018). 30194396 10.1038/s41467-018-05428-6 PMC6128903

[21] Lustbader, E. D., Williams, W. R., Bondy, M. L., Strom, S. & Strong, L. C. Segregation analysis of cancer in families of childhood soft-tissue-sarcoma patients. *Am. J. Hum. Genet*. **51**, 344–356 (1992). 1642235 PMC1682662

[22] Bougeard, G. et al. Revisiting Li-Fraumeni syndrome from TP53 mutation carriers. *J. Clin. Oncol.* **33**, 2345–2352 (2015). 26014290 10.1200/JCO.2014.59.5728

[23] Sorrell, A. D., Espenschied, C. R., Culver, J. O. & Weitzel, J. N. Tumor protein p53 (TP53) testing and Li-Fraumeni syndrome: current status of clinical applications and future directions. *Mol. Diagn. Ther.* **17**, 31–47 (2013). 23355100 10.1007/s40291-013-0020-0 PMC3627545

[24] Bondy, M. L. et al. Brain tumor epidemiology: consensus from the Brain Tumor Epidemiology Consortium. *Cancer* **113**, 1953–1968 (2008). 18798534 10.1002/cncr.23741 PMC2861559

[25] Ostrom, Q. T. et al. The epidemiology of glioma in adults: a “state of the science” review. *Neuro Oncol.* **16**, 896–913 (2014). 24842956 10.1093/neuonc/nou087 PMC4057143

[26] McCarthy, S. et al. A reference panel of 64,976 haplotypes for genotype imputation. *Nat. Genet.* **48**, 1279–1283 (2016). 27548312 10.1038/ng.3643 PMC5388176

[27] Howie, B., Fuchsberger, C., Stephens, M., Marchini, J. & Abecasis, G. R. Fast and accurate genotype imputation in genome-wide association studies through pre-phasing. *Nat. Genet*. **44**, 955 (2012). 22820512 10.1038/ng.2354 PMC3696580

[28] Marchini, J. & Howie, B. Genotype imputation for genome-wide association studies. *Nat. Rev. Genet.* **11**, 499 (2010). 20517342 10.1038/nrg2796

[29] Yamada, H. et al. Identification and characterization of a novel germline p53 mutation in a patient with glioblastoma and colon cancer. *Int J. Cancer* **125**, 973–976 (2009). 19405127 10.1002/ijc.24432

[30] Birch, J. M. et al. Relative frequency and morphology of cancers in carriers of germline TP53 mutations. *Oncogene* **20**, 4621–4628 (2001). 11498785 10.1038/sj.onc.1204621

[31] Donehower, L. A. & Lozano, G. 20 years studying p53 functions in genetically engineered mice. *Nat. Rev. Cancer* **9**, 831–841 (2009). 19776746 10.1038/nrc2731

[32] Joerger, A. C. & Fersht, A. R. The tumor suppressor p53: from structures to drug discovery. *Cold Spring Harb. Perspect. Biol.* **2**, a000919 (2010). 20516128 10.1101/cshperspect.a000919 PMC2869527

[33] Raj, N. & Attardi, L. D. The transactivation domains of the p53 protein. *Cold Spring Harb. Perspect. Med.* **7**, a026047 (2017). 10.1101/cshperspect.a026047 PMC5204331 27864306

[34] Verhaak, R. G. et al. Integrated genomic analysis identifies clinically relevant subtypes of glioblastoma characterized by abnormalities in PDGFRA, IDH1, EGFR, and NF1. *Cancer Cell* **17**, 98–110 (2010). 20129251 10.1016/j.ccr.2009.12.020 PMC2818769

[35] Lei, L. et al. Glioblastoma models reveal the connection between adult glial progenitors and the proneural phenotype. *PloS ONE* **6**, e20041 (2011). 21625383 10.1371/journal.pone.0020041 PMC3100315

[36] Guy, C. T., Cardiff, R. D. & Muller, W. J. Induction of mammary tumors by expression of polyomavirus middle T oncogene: a transgenic mouse model for metastatic disease. *Mol. Cell Biol.* **12**, 954–961 (1992). 1312220 10.1128/mcb.12.3.954-961.1992 PMC369527

[37] Wilson, J. R. et al. A novel HER2-positive breast cancer phenotype arising from germline TP53 mutations. *J. Med. Genet.* **47**, 771–774 (2010). 20805372 10.1136/jmg.2010.078113

[38] Fletcher, O. & Houlston, R. S. Architecture of inherited susceptibility to common cancer. *Nat. Rev. Cancer* **10**, 353–361 (2010). 20414203 10.1038/nrc2840

[39] Hindorff, L. A., Gillanders, E. M. & Manolio, T. A. Genetic architecture of cancer and other complex diseases: lessons learned and future directions. *Carcinogenesis* **32**, 945–954 (2011). 21459759 10.1093/carcin/bgr056 PMC3140138

[40] Hoffman, A. E. et al. microRNA miR-196a-2 and breast cancer: a genetic and epigenetic association study and functional analysis. *Cancer Res.* **69**, 5970–5977 (2009). 19567675 10.1158/0008-5472.CAN-09-0236 PMC2716085

[41] Pongsavee, M. et al. The BRCA1 3’-UTR: 5711+421T/T_5711+1286T/T genotype is a possible breast and ovarian cancer risk factor. *Genet Test. Mol. Biomark.* **13**, 307–317 (2009). 10.1089/gtmb.2008.0127 19405875

[42] Tchatchou, S. et al. A variant affecting a putative miRNA target site in estrogen receptor (ESR) 1 is associated with breast cancer risk in premenopausal women. *Carcinogenesis* **30**, 59–64 (2009). 19028706 10.1093/carcin/bgn253

[43] Chin, L. J. et al. A SNP in a let-7 microRNA complementary site in the KRAS 3′ untranslated region increases non-small cell lung cancer risk. *Cancer Res*. **68**, 8535–8540 (2008). 18922928 10.1158/0008-5472.CAN-08-2129 PMC2672193

[44] Ratner, E. et al. A KRAS-variant in ovarian cancer acts as a genetic marker of cancer risk. *Cancer Res.* **70**, 6509–6515 (2010). 20647319 10.1158/0008-5472.CAN-10-0689 PMC2923587

[45] Paranjape, T. et al. A 3’-untranslated region KRAS variant and triple-negative breast cancer: a case-control and genetic analysis. *Lancet Oncol.* **12**, 377–386 (2011). 21435948 10.1016/S1470-2045(11)70044-4 PMC3488438

[46] Hollestelle, A. et al. No clinical utility of KRAS variant rs61764370 for ovarian or breast cancer. *Gynecol. Oncol.* **141**, 386–401 (2016). 25940428 10.1016/j.ygyno.2015.04.034 PMC4630206

[47] Wu, C. C., Shete, S., Amos, C. I. & Strong, L. C. Joint effects of germ-line p53 mutation and sex on cancer risk in Li-Fraumeni syndrome. *Cancer Res*. **66**, 8287–8292 (2006). 16912210 10.1158/0008-5472.CAN-05-4247

[48] Samuel, N. et al. Genome-wide DNA methylation analysis reveals epigenetic dysregulation of MicroRNA-34A in TP53-associated cancer susceptibility. *J. Clin. Oncol.* **34**, 3697–3704 (2016). 27551116 10.1200/JCO.2016.67.6940 PMC6366343

[49] Kratz, C. P. et al. Cancer screening recommendations for individuals with Li-Fraumeni syndrome. *Clin. Cancer Res.* **23**, e38–e45 (2017). 28572266 10.1158/1078-0432.CCR-17-0408

[50] Guan, X., Wang, L. E., Liu, Z., Sturgis, E. M. & Wei, Q. Association between a rare novel TP53 variant (rs78378222) and melanoma, squamous cell carcinoma of head and neck and lung cancer susceptibility in non-Hispanic Whites. *J. Cell Mol. Med.* **17**, 873–878 (2013). 23742673 10.1111/jcmm.12076 PMC3729608

[51] Fumagalli, S. & Thomas, G. The role of p53 in Ribosomopathies. *Semin Hematol.* **48**, 97–105 (2011). 21435506 10.1053/j.seminhematol.2011.02.004

[52] Van Nostrand, J. L. et al. Inappropriate p53 activation during development induces features of CHARGE syndrome. *Nature* **514**, 228–232 (2014). 25119037 10.1038/nature13585 PMC4192026

[53] Faustino-Rocha, A. et al. Estimation of rat mammary tumor volume using caliper and ultrasonography measurements. *Lab Anim.* **42**, 217–224 (2013). 10.1038/laban.254 23689461

[54] Kitamura, T. et al. CCL2-induced chemokine cascade promotes breast cancer metastasis by enhancing retention of metastasis-associated macrophages. *J. Exp. Med.* **212**, 1043–1059 (2015). 26056232 10.1084/jem.20141836 PMC4493415

[55] Gensert, J. M. & Goldman, J. E. Heterogeneity of cycling glial progenitors in the adult mammalian cortex and white matter. *J. Neurobiol.* **48**, 75–86 (2001). 11438938

[56] Annunziato, S. et al. Modeling invasive lobular breast carcinoma by CRISPR/Cas9-mediated somatic genome editing of the mammary gland. *Genes Dev.* **30**, 1470–1480 (2016). 27340177 10.1101/gad.279190.116 PMC4926868

[57] Du, Z. et al. Introduction of oncogenes into mammary glands in vivo with an avian retroviral vector initiates and promotes carcinogenesis in mouse models. *Proc. Natl Acad. Sci. USA* **103**, 17396–17401 (2006). 17090666 10.1073/pnas.0608607103 PMC1635021

[58] Krause, S., Brock, A. & Ingber, D. E. Intraductal injection for localized drug delivery to the mouse mammary gland. *J. Vis. Exp.* **80**, e50692 (2013). 10.3791/50692 PMC3938324 24121742

[59] Ni, T. et al. Snail1-dependent p53 repression regulates expansion and activity of tumour-initiating cells in breast cancer. *Nat. Cell Biol.* **18**, 1221–1232 (2016). 27749822 10.1038/ncb3425 PMC6038146

[60] Plante, I., Stewart, M. K. & Laird, D. W. Evaluation of mammary gland development and function in mouse models. *J. Vis. Exp.* **53**, e2828 (2011). 10.3791/2828 PMC3196158 21808224

[61] Fierz, Y., Novosyadlyy, R., Vijayakumar, A., Yakar, S. & LeRoith, D. Insulin-sensitizing therapy attenuates type 2 diabetes-mediated mammary tumor progression. *Diabetes* **59**, 686–693 (2010). 19959755 10.2337/db09-1291 PMC2828655

[62] Rueden, C. T. et al. ImageJ2: ImageJ for the next generation of scientific image data. *BMC Bioinform.* **18**, 529 (2017). 10.1186/s12859-017-1934-z PMC5708080 29187165

[63] Agarwal, V., Bell, G. W., Nam, J. W. & Bartel, D. P. Predicting effective microRNA target sites in mammalian mRNAs. *eLife* **4**, e05005 (2015). 10.7554/eLife.05005 PMC4532895 26267216

[64] Bhattacharya, A., Ziebarth, J. D. & Cui, Y. PolymiRTS database 3.0: linking polymorphisms in microRNAs and their target sites with human diseases and biological pathways. *Nucl. Acids Res.* **42**, D86–D91 (2014). 24163105 10.1093/nar/gkt1028 PMC3965097

[65] Birch, J. M. et al. Prevalence and diversity of constitutional mutations in the p53 gene among 21 Li-Fraumeni families. *Cancer Res.* **54**, 1298–1304 (1994). 8118819

[66] Eeles, R. A. Germline mutations in the TP53 gene. *Cancer Surv.* **25**, 101–124 (1995). 8718514

[67] McBride, K. A. et al. Li-Fraumeni syndrome: cancer risk assessment and clinical management. *Nat. Rev. Clin. Oncol.* **11**, 260–271 (2014). 24642672 10.1038/nrclinonc.2014.41

