# Supplementary material for: Tissue-specific microRNA expression alters cancer susceptibility conferred by a TP53 noncoding variant
Source: Nat Commun. 2019 Nov 7;10:5061. doi: 10.1038/s41467-019-13002-x (PMC6838078; doi:10.1038/s41467-019-13002-x)
Supplement: Supplementary file 1 — Supplementary Information [file 41467_2019_13002_MOESM1_ESM.pdf]

Supplementary Materials for

"Tissue-specific MicroRNA Expression Alters Cancer Susceptibility Conferred by a *TP53* Noncoding Variant"

Deng *et al*.

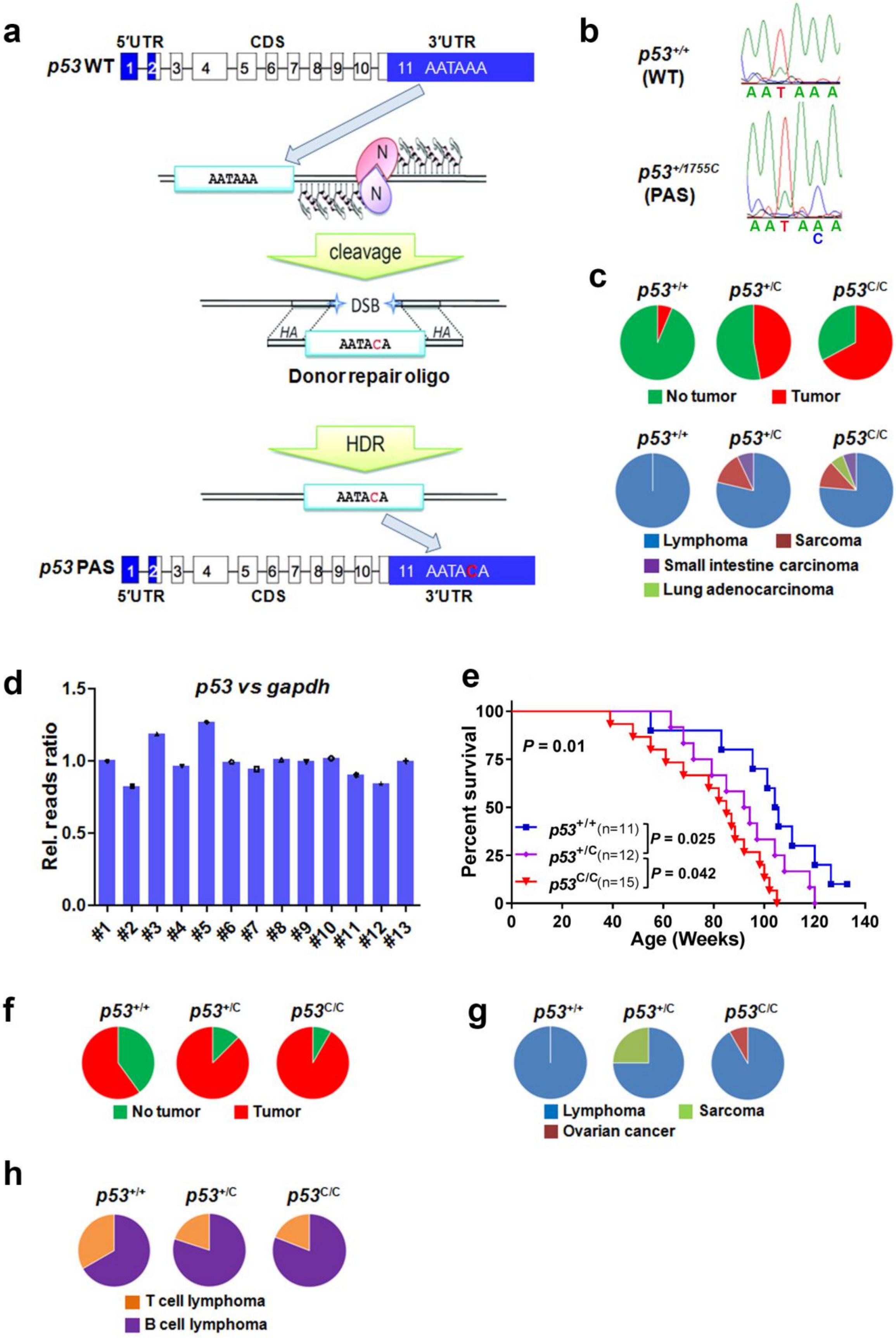
a
5′UTR
CDS
3′UTR
p53 WT
1
2
3
4
5
6
7
8
9
10
11 AATAAA
AATAAA
N
N
cleavage
DSB
HA
HA
AATACA
Donor repair oligo
HDR
AATACA
p53 PAS
11 AATACA
b
p53+/+
(WT)
A A T A A A
p53+/1755C
(PAS)
A A T A A A
C
c
p53+/+
p53+/C
p53C/C
No tumor
Tumor
Lymphoma
Sarcoma
Small intestine carcinoma
Lung adenocarcinoma
d
p53 vs gapdh
Rel. reads ratio
1.5
1.0
0.5
0.0
#1 #2 #3 #4 #5 #6 #7 #8 #9 #10 #11 #12 #13
e
Percent survival
100
75
50
25
0
P = 0.01
p53+/+(n=11)
p53+/C(n=12)
p53C/C(n=15)
P = 0.025
P = 0.042
0 20 40 60 80 100 120 140
Age (Weeks)
f
p53+/+
p53+/C
p53C/C
No tumor
Tumor
g
p53+/+
p53+/C
p53C/C
Lymphoma
Sarcoma
Ovarian cancer
h
p53+/+
p53+/C
p53C/C
T cell lymphoma
B cell lymphoma

Supplementary Fig. 1. The *p53*^{C} allele accelerates spontaneous tumorigenesis and radiation-induced tumorigenesis in mice. (**a**) Schematic diagram showing the mutation at the endogenous *p53* gene polyadenylation signal (PAS) with zinger finger nuclease-mediated cleavage and homology-directed repair. Numbers in rectangles indicate exons of the *p53* gene; blue rectangles indicate untranslated regions (UTR) and clear rectangles indicate coding sequences. Lines connecting rectangles indicate introns in the *p53* gene. (**b**) Confirmation of the mutation introduction by DNA sequencing. (**c**) Proportions of *p53*$^{+/+}$, *p53*$^{+/C}$, and *p53*$^{C/C}$ littermates that developed spontaneous tumors during their lifespan; tumor types were shown. (**d**)The relative read ratio (Rel. read ratio) for *p53* normalized to that of *Gapdh* in genomic DNA isolated from B cell lymphoma that developed in mutant mice, as determined by exome sequencing. (**e**) Survival of *p53*$^{+/+}$, *p53*$^{+/C}$, and *p53*$^{C/C}$ littermates treated with a single dose of 4 Gy γ-irradiation at 6 weeks of age. $P = 0.01$ (log-rank test). (**f**) Proportions of mutant and WT littermates that developed tumors during their lifetimes after irradiation. (**g**) Cancer spectrum of irradiated mice. (**h**) Proportions of T cell lymphoma and B cell lymphoma in irradiated mice.

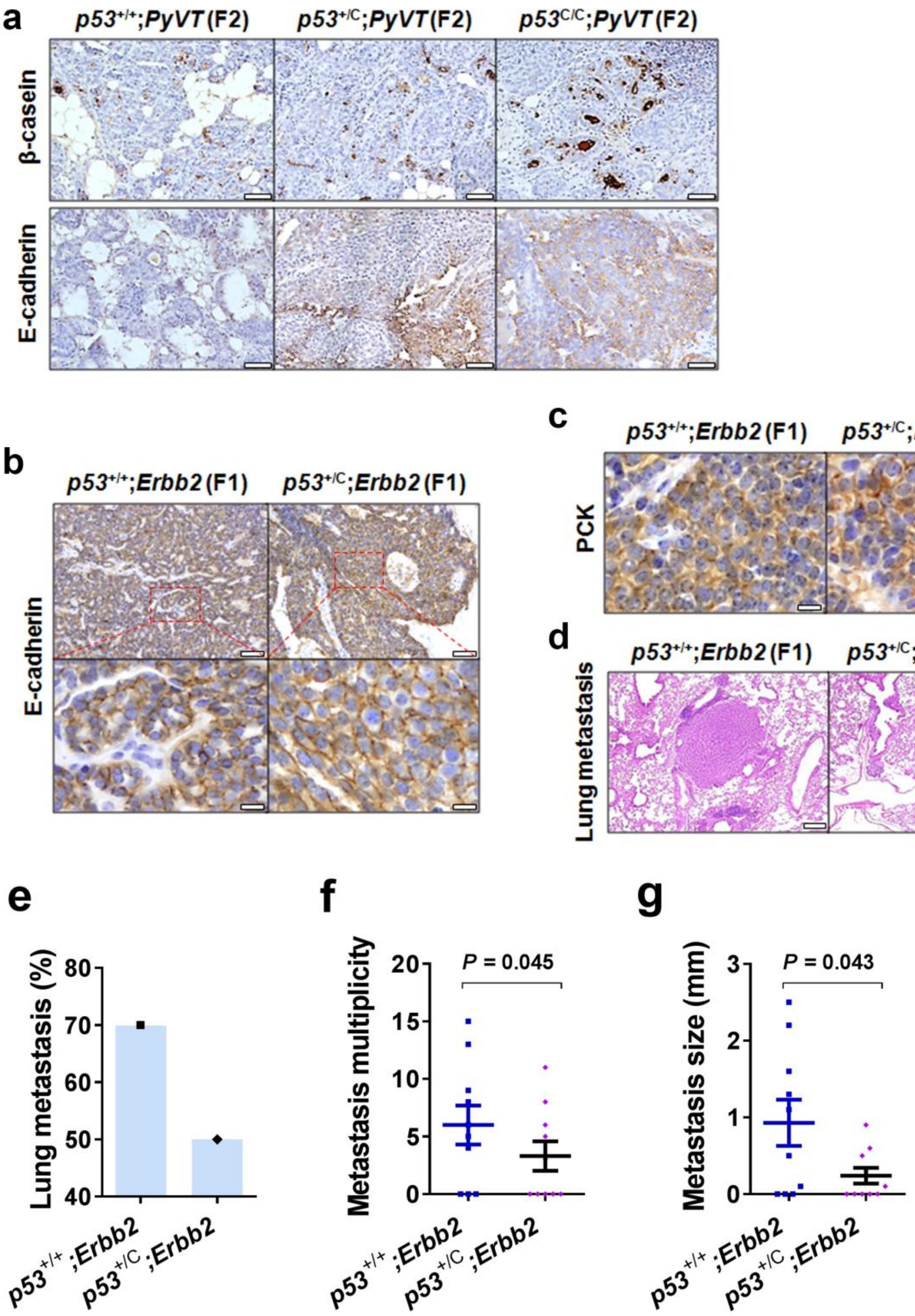
a
p53+/+;PyVT (F2)
p53+/C;PyVT (F2)
p53C/C;PyVT (F2)
β-casein
E-cadherin
b
p53+/+;Erbb2 (F1)
p53+/C;Erbb2 (F1)
E-cadherin
c
p53+/+;Erbb2 (F1)
p53+/C;Erbb2 (F1)
PCK
d
p53+/+;Erbb2 (F1)
p53+/C;Erbb2 (F1)
Lung metastasis
e
Lung metastasis (%)
80
70
60
50
40
p53+/+;Erbb2
p53+/C;Erbb2
f
Metastasis multiplicity
P = 0.045
20
15
10
5
0
p53+/+;Erbb2
p53+/C;Erbb2
g
Metastasis size (mm)
P = 0.043
3
2
1
0
p53+/+;Erbb2
p53+/C;Erbb2

Supplementary Fig. 2. The *p53*^{C} allele delays mammary tumor progression and metastasis in mice. (**a**) Immunohistochemistry (IHC) staining of β-casein and E-cadherin in tumors from 12-week-old F2 *PyVT* females. $n = 3$ mice per group. Scale bar, 50 µm. (**b**) IHC staining for E-cadherin in mammary tumors from F1 *p53*$^{+/+}$;*Erbb2* mice and *p53*$^{+/C}$;*Erbb2* mice. Scale bar, 100 µm (top row), 20 µm (bottom row). $n \geq 3$ tumors for each genotype (one tumor per animal). (**c**) IHC staining for the epithelial marker pan cytokeratin (PCK) in mammary tumors from F1 *p53*$^{+/+}$;*Erbb2* and *p53*$^{+/C}$;*Erbb2* mice. Scale bar, 20 µm. $n \geq 3$ tumors for each genotype (one tumor per animal). (**d**) Representative H&E staining for lung metastasis arising from mammary tumors in *p53*$^{+/+}$;*Erbb2* and *p53*$^{+/C}$;*Erbb2* mice. Scale bar, 200 µm. (**e**) Percentage of animals with lung metastasis. (**f**) Lung metastasis multiplicity. $P = 0.045$ (Mann-Whitney U test). Error bars depict s.e.m. (**g**) Lung metastatic tumor size. The average size of nodules (total nodule area [mm2]/total nodule number) was determined and converted to the average diameter (mm). $n = 10$ mice for each genotype. $P = 0.043$ (Mann-Whitney U test). Error bars depict s.e.m.

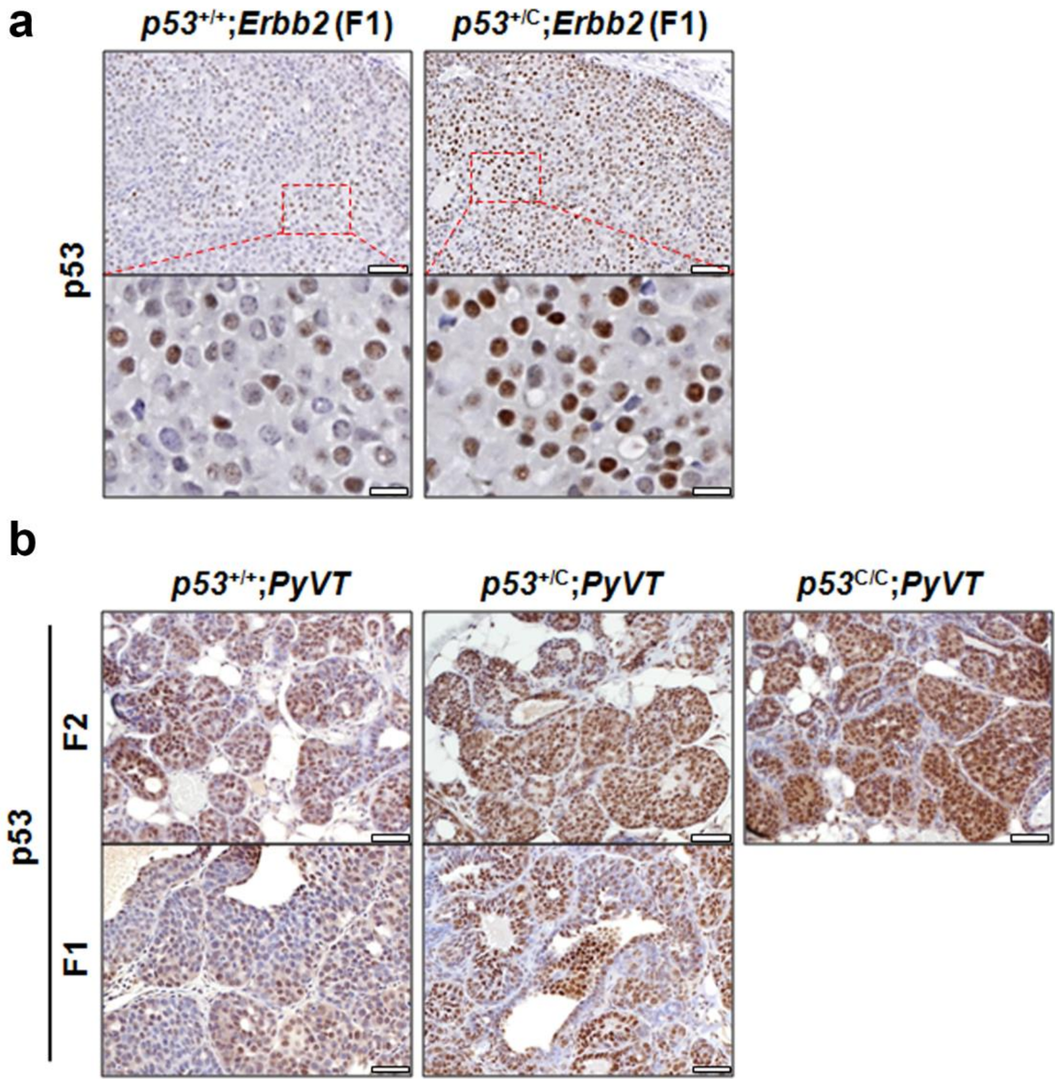

Supplementary Fig. 3. p53 protein levels in mammary tumors that developed in mice. (**a**) IHC staining for p53 protein levels in mammary tumors from *p53*$^{+/+}$;*Erbb2* and *p53*$^{+/C}$;*Erbb2* mice. Scale bar, 100 μm (top row), 20 μm (bottom row). $n \geq 3$ tumors for each genotype. (**b**) IHC staining for p53 protein levels in mammary tumors from *p53*$^{+/+}$;*PyVT*, *p53*$^{+/C}$;*PyVT* and *p53*$^{C/C}$;*PyVT* mice, either F2 or F1. Scale bar, 50 μm. $n \geq 3$ tumors for each genotype.

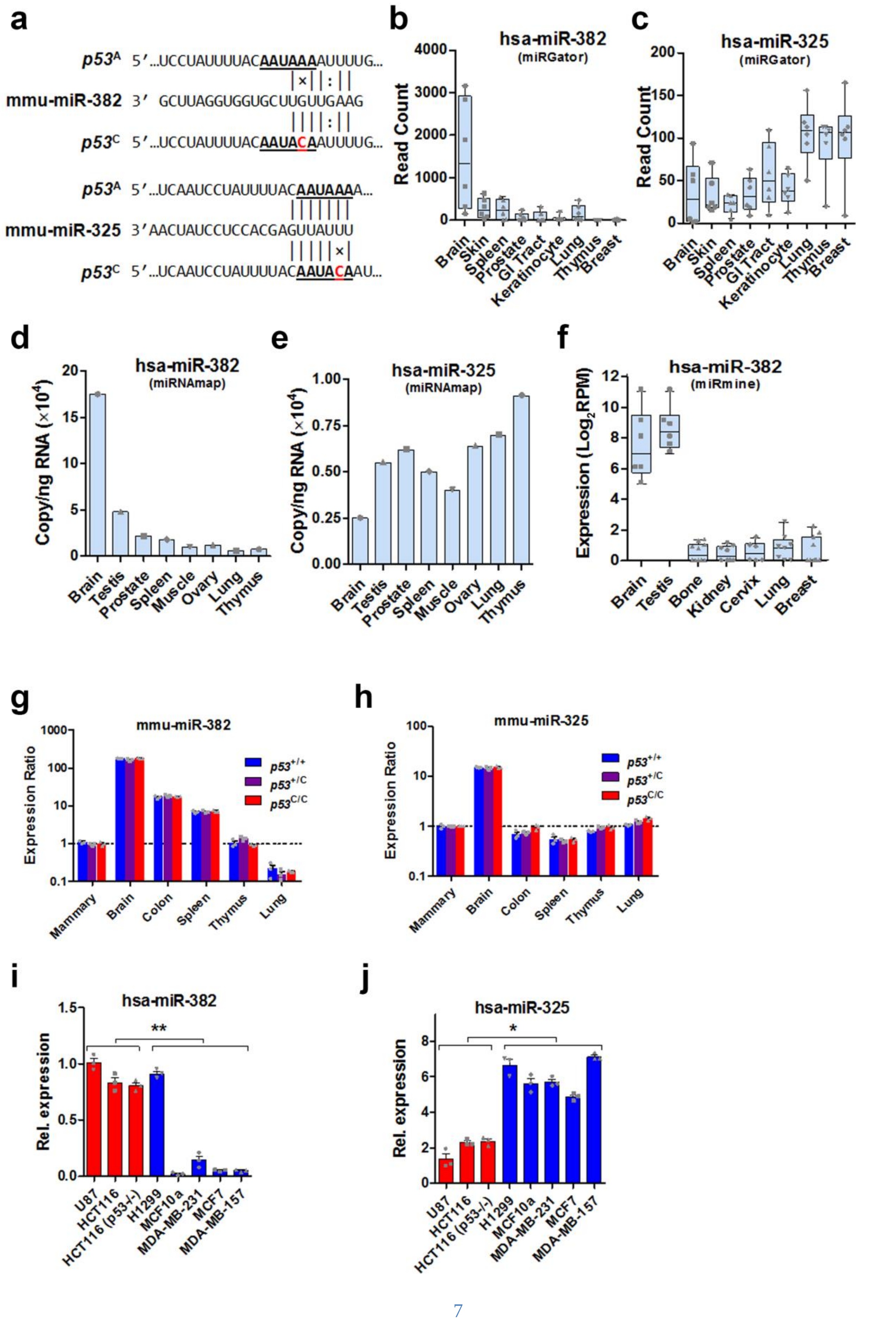
a
p53A 5′...UCCUAUUUUACAAUAAAAUUUUG...
mmu-miR-382 3′ GCUUAGGUGGUGCUUGUUGAAG
p53C 5′...UCCUAUUUUACAAUACAAUUUUG...
p53A 5′...UCAAUCCUAUUUUACAAUAAAA...
mmu-miR-325 3′AACUAUCCUCCACGAGUUAUUU
p53C 5′...UCAAUCCUAUUUUACAAUACAAU...
b
hsa-miR-382
(miRGator)
Read Count
Brain
Skin
Spleen
Prostate
GI Tract
Keratinocyte
Lung
Thymus
Breast
c
hsa-miR-325
(miRGator)
Read Count
d
hsa-miR-382
(miRNAmap)
Copy/ng RNA (×10^4)
Brain
Testis
Prostate
Spleen
Muscle
Ovary
Lung
Thymus
e
hsa-miR-325
(miRNAmap)
Copy/ng RNA (×10^4)
f
hsa-miR-382
(miRmine)
Expression (Log2RPM)
Brain
Testis
Bone
Kidney
Cervix
Lung
Breast
g
mmu-miR-382
Expression Ratio
p53+/+
p53+/C
p53C/C
Mammary
Brain
Colon
Spleen
Thymus
Lung
h
mmu-miR-325
Expression Ratio
i
hsa-miR-382
Rel. expression
**
U87
HCT116
HCT116 (p53-/-)
H1299
MCF10a
MDA-MB-231
MCF7
MDA-MB-157
j
hsa-miR-325
Rel. expression
*

Supplementary Fig. 4. miR-325 and miR-382 expression is tissue-specific. (**a**) Schematic diagram showing the creation of a miR-382 targeting site and the disruption of a miR-325 targeting site in mouse *p53* 3′UTR due to the $p53^{C}$ allele. (**b** and **c**) Expression levels (read count) of miR-382 (**b**) and miR-325 (**c**) in different human tissues by deep sequencing. Data were obtained from the online database miRGator. (**d** and **e**) Expression level (copy number) of miR-382 (**d**) and miR-325 (**e**) in indicated human tissues as determined by deep sequencing. Data were extracted from the online database miRNAmap, which contained no data on the expression of miR-382 and miR-325 in breast tissue. (**f**) Expression level of miR-382 in indicated human tissues. Data were extracted from the miRmine database, which contained no miR-325 expression data; $\log_2$RPM, represents the logarithm of the number of miRNA reads per million total RNA reads to the base 2. (**g**) Relative expression of miR-382 in indicated tissues from $p53^{+/+}$, $p53^{+/C}$ and $p53^{C/C}$ mice measured by qPCR. $n = 3$ mice, each performed with technical triplicates. (**h**) Relative expression of miR-325 in indicated tissues from $p53^{+/+}$, $p53^{+/C}$ and $p53^{C/C}$ mice measured by qPCR. $n = 3$ mice. Please note the differences in scales between **g** and **h**. (**i** and **j**) qPCR analysis for miR-382 (**i**) and miR-325 (**j**) levels in human cancer cell lines. Rel. expression: Relative expression of miRNAs normalized to U6 RNA. Red columns indicate cell lines established from human glioma and colon cancers, and blue columns indicate cell lines established from human breast and lung cancers. $n = 3$ independent biological replications with technical triplicates. * $P < 0.05$, ** $P < 0.01$ (one-way ANOVA). Error bars depict s.e.m.

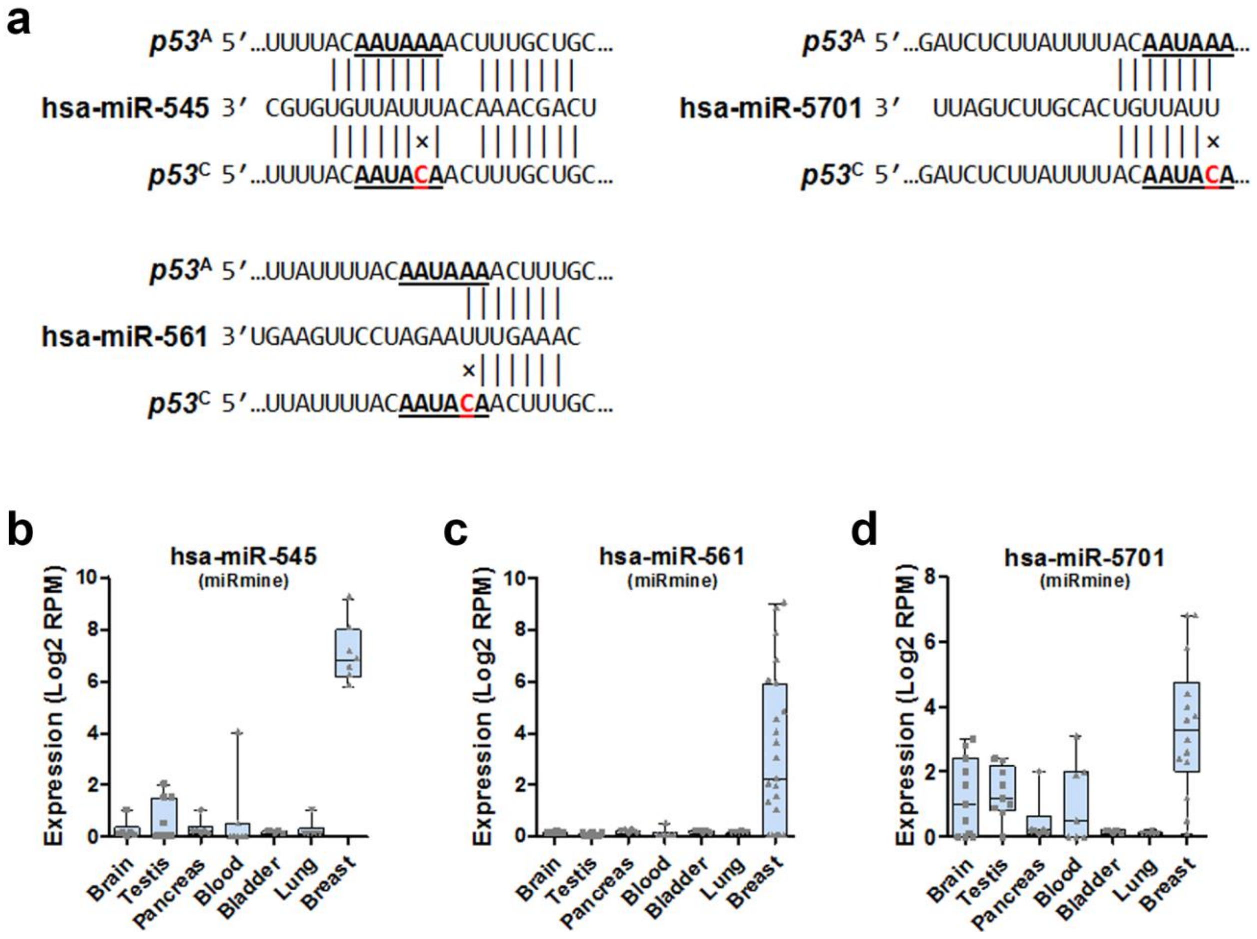

Supplementary Fig. 5. The expression of other miRNAs targeting the WT *TP53* 3′UTR. (**a**) Schematic diagram showing the disruption of miR-545, miR-561, and miR-5701 targeting sites in human *TP53* 3′UTR due to the PAS variant. (**b-d**) Expression level of miR-545 (**b**), miR-561 (**c**) and miR-5701 (**d**) in indicated human tissues as determined by deep sequencing. Data were extracted from the online database miRmine.

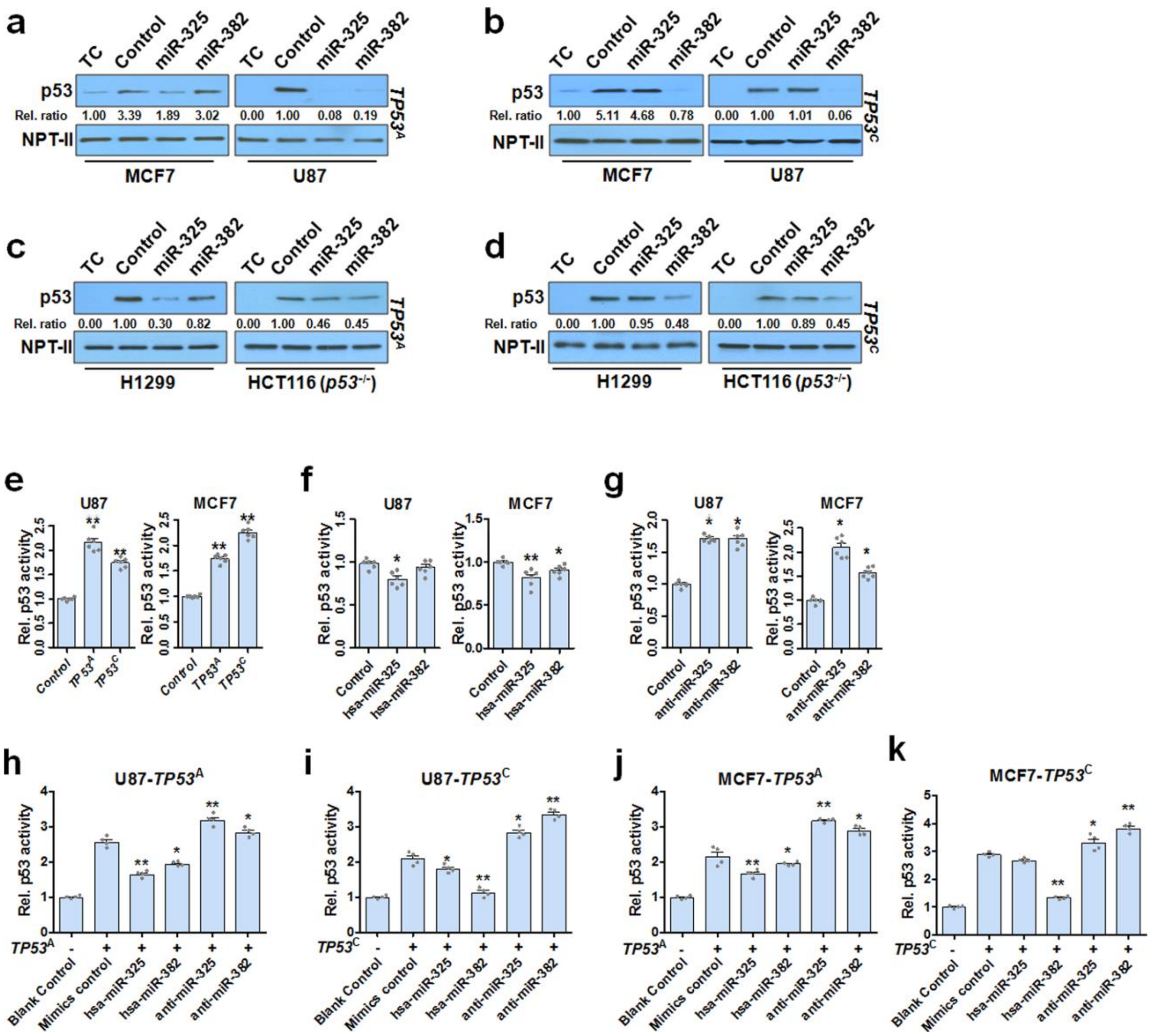

Supplementary Fig. 6. miRNA suppression of p53 expression and p53-driven luciferase activity. a-d, Western blotting analysis for p53 expression in several cell lines transfected with a p53-expressing plasmid with WT or the variant 3′UTR (*TP53*^{A} or *TP53*^{C}) and miR-325 or miR-382 mimics. Source data are provided in a Source Data file. Control, transfection with the *TP53*^{A} or *TP53*^{C} with miRNA mimics negative control. Rel. ratio: the relative signal densities of p53 as referenced to that of NPT-II. $n \geq 3$ experiments for each transfection and western blotting. (**a**) MCF7 and U87 cells transfected *TP53*^{A} and miR-325 or miR-382. (**b**) MCF7 and U87 cells transfected with *TP53*^{C} and miR-325 or miR-382. (**c**) H1299 and HCT116 (*TP53*$^{-/-}$) cells transfected with *TP53*^{A} and miR-325 or miR-382. (**d**) H1299 and HCT116 (*TP53*$^{-/-}$) cells transfected with *TP53*^{C} and miR-325 or miR-382. TC = transfection control. **e-k,** NanoLuc

luciferase assays for p53 transcriptional activity in U87 and MCF7 cells. All cells were transfected with pNL(NLucP/p53-RE/Hygro) (Promega), along with an exogenous p53-expressing plasmid with a native 3′UTR (*TP53*^{A}) or a 3′UTR with an alternative PAS (*TP53*^{C}) (**e**), miRNA mimics (**f**), or anti-miRNAs (**g**), or a combination of p53 expression plasmid and miRNA mimics (or anti-miRNAs) (**h**-**k**). Rel. p53 activity: relative p53 activity as determined by relative luminance units. Either endogenous or exogenous p53 drives NlucP expression. Data were presented as mean ±s.e.m; error bars depict s.e.m. n = 3-6 for each group and n = 3 for biological replications. * $P < 0.05$, **, $P < 0.01$.

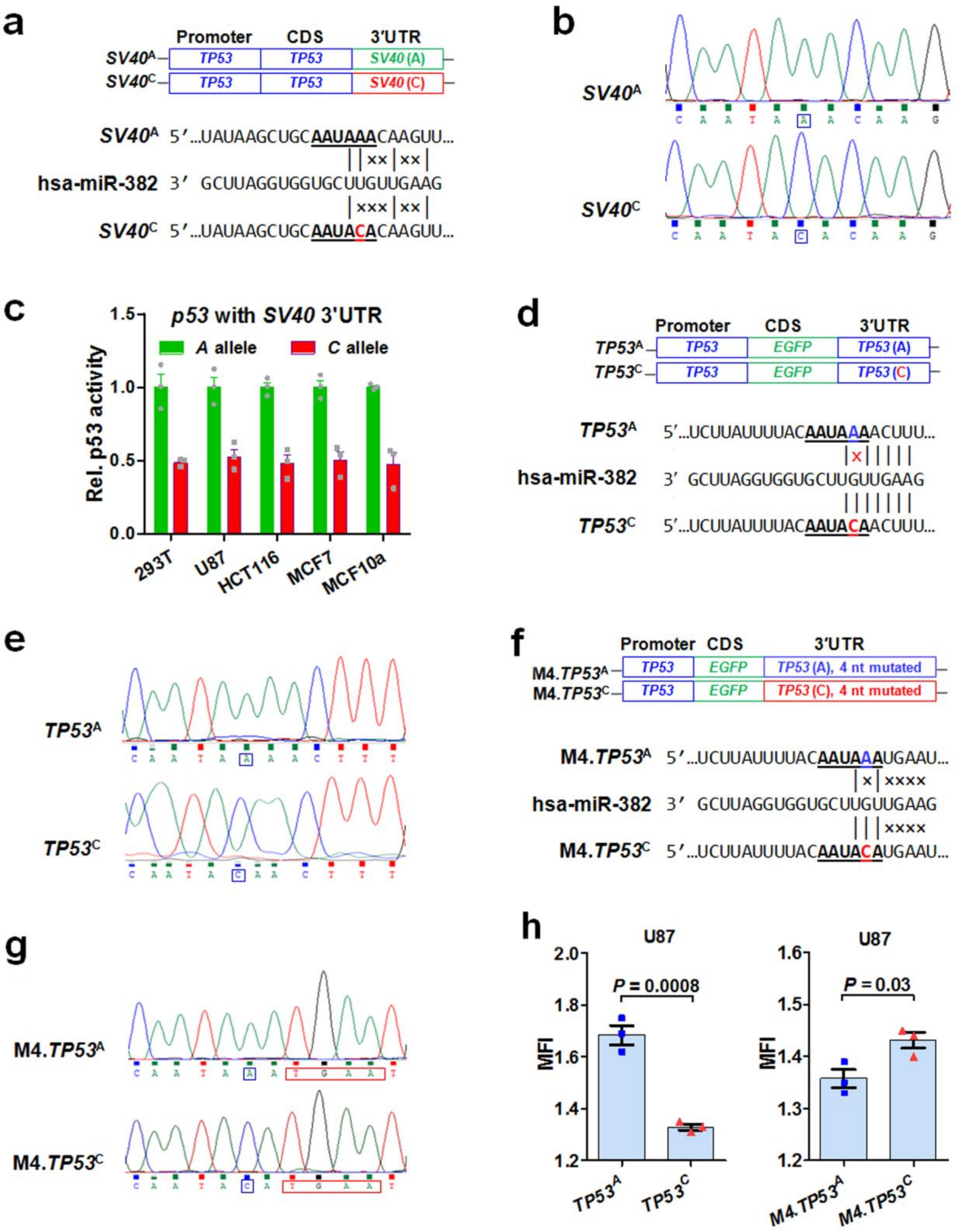

Supplementary Fig. 7. The integrity of the miR-382 binding sites in miR-382-mediated *TP53*^{C} suppression. (**a**) Schematic diagram and (b) Sanger DNA sequencing showing p53 expression plasmids with SV40 3′UTR WT PAS [*SV40* (A)] or the PAS variant [*SV40* (C)] and

the absence of the miR-382 targeting site in these sequences. (**c**) NanoLuc luciferase assay for p53 transcriptional activity in indicated cell lines transfected with plasmids shown in (**a**). Rel. p53 activity: relative p53 activity as determined by relative luminance units, each normalized to cells with the *A* allele. Data were presented as mean + s.e.m; error bars depict s.e.m. $n = 3$ for each group. (**c**) Schematic diagram showing EGFP expression plasmids with *p53* 3′UTR with WT PAS [*TP53* (A)] or the variant PAS [(*TP53* (C)] and the presence of miR-382 targeting sites (left). The mutation of 4 nucleotides (M4) in *TP53* 3′UTR with WT PAS [M4.*TP53* (A)] or a PAS variant [M4.*TP53* (C)] eliminated the miR-382 targeting site (right). (**d**) Schematic diagram and (**e**) sequencing showing EGFP expression plasmids with WT *p53* 3′UTR ($TP53^{A}$) or the *TP53* 3′UTR variant ($TP53^{C}$) and the presence of miR-382 targeting sites. (**f, g**) The mutation of 4 nucleotides (M4) in WT *TP53* 3′UTR (M4.$TP53^{A}$) or the *TP53* 3′UTR variant (M4.$TP53^{C}$) eliminated the miR-382 targeting site. (**h**) EGFP expression in U87 cells transfected with the 4 plasmids constructed in (**d** and **f**). An RFP expression plasmid with the native *SV40* 3′UTR with no miR-382 sites was co-transfected as a normalization control. Y axis denotes the MFI (mean fluorescence intensity) for EGFP normalized to that of RFP. $n = 3$ biological triplicates and $n = 3$ technical triplicates.

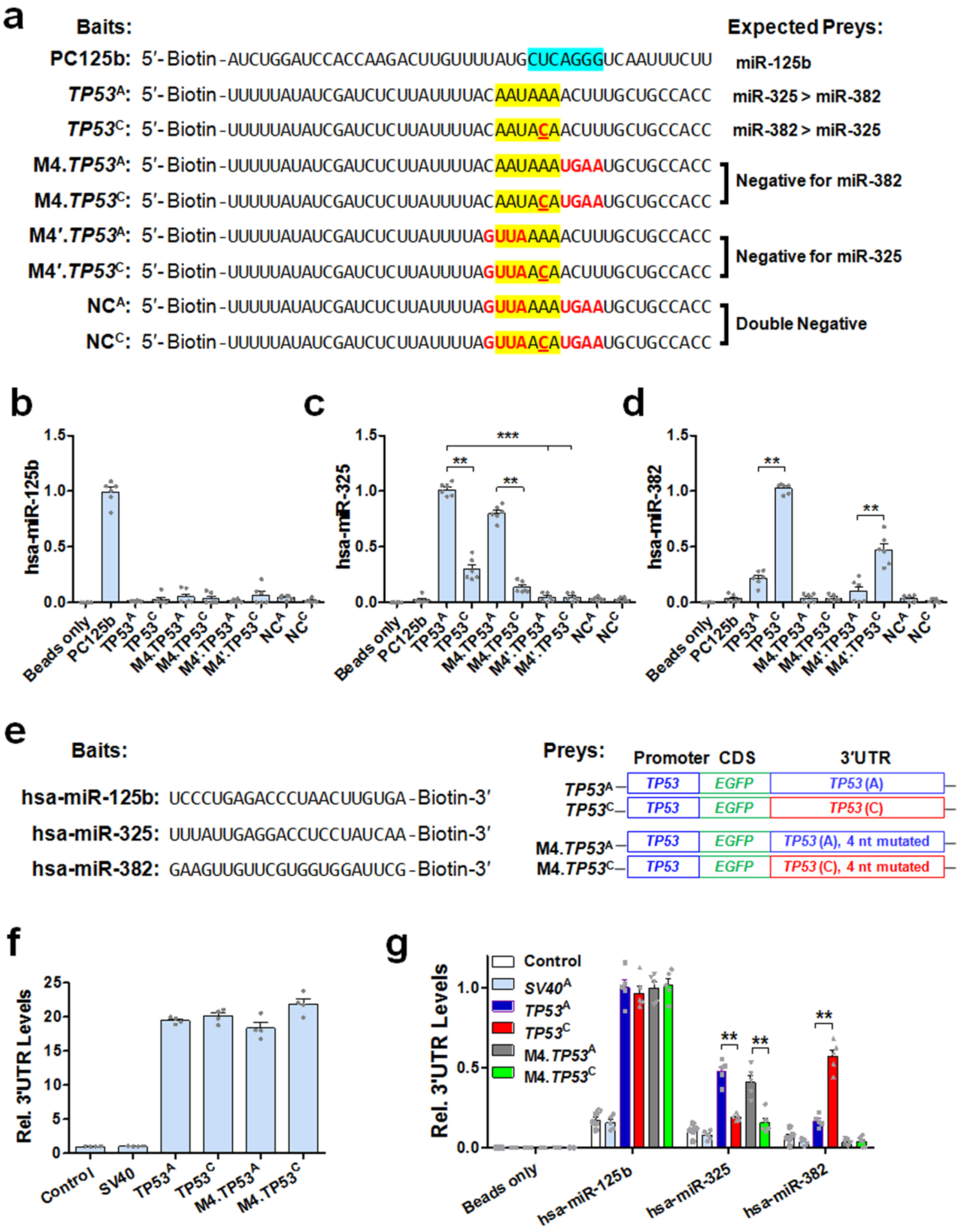

Supplementary Fig. 8. Direct binding of *TP53* 3′UTR and miRNAs using pull-down assays.

(**a**) Schematic diagram showing 5′end biotin-labeled *TP53* 3'UTR RNA fragments. Blue block,

sequence complementary to miR-125b seed; yellow block, PAS; bold letters in red, mutated nucleotides to remove miR-325/miR-382 sites. PC125b: RNA fragment of *TP53* 3′UTR containing miR-125b site; $TP53^{A}$: RNA fragment of *TP53* 3′UTR containing WT PAS (*A* allele); $TP53^{C}$: same as $TP53^{A}$ except the alternative PAS (*C* allele); M4.$TP53^{A}$: same as $TP53^{A}$ except 4 nucleotides mutated to eliminate miR-382 site; M4.$TP53^{C}$: same as $TP53^{C}$ except 4 nucleotides mutated to eliminate miR-382 site; M4′.$TP53^{A}$: same as $TP53^{A}$ except 4 nucleotides mutated to eliminate miR-325 site; M4′.$TP53^{C}$: same as $TP53^{C}$ except 4 nucleotides mutated to eliminate miR-325 site; M8.$TP53^{A}$: same as $TP53^{A}$ except 8 nucleotides mutated to eliminate both sites; M8.$TP53^{C}$: same as $TP53^{C}$ except 8 nucleotides mutated to eliminate both sites. Biotinylated RNA fragments were transfected into H1299 cells. Cells after 24h were lysed followed by Pierce™ Streptavidin Magnetic Beads incubation and pull-down. After DNase I treatment, RNA purification, and reverse transcription, the relative amount of co-purified miR-125b (**b**), miR-325 (**c**) and miR-382 (**d**) were determined by qPCR. Values were normalized to the sample with the highest amount of miRNAs. Error bars depict s.e.m. $n = 3$ biological triplicates and $n = 4$-6 technical triplicates. **, $P < 0.01$; ***, $P < 0.001$ (one-way ANOVA). (**e**) Schematic diagram showing 3′end biotin-labeled miRNAs and the 4 constructs expressing EGFP with *TP53* 3′UTR (Supplementary Fig. 7). **(f,g)** H1299 cells were mock transfected (Control), transfected with a luciferase report plasmid with *SV40* 3′UTR, transfected an EGFP expression plasmid, or co-transfected with an EGFP expression plasmid and a biotinylated miRNA (**e**). Cells were lysed 24h after transfection, miRNA-interacting partners purified, and *TP53* 3′UTR levels determined by qPCR. Values were normalized to that of miR-125b in cells with $TP53^{A}$. Error bars depict s.e.m. $n = 3$ biological triplicates and $n = 3$-6 technical triplicates. ** $P < 0.01$ (two-sided Student’s t-test.).

Supplementary Table 1. rs78378222[C] and cancer risk

Cancers	MAF[1] (%)	Cases (N)	Controls (N)	OR[2]	*P* Value	References
Glioma	1.3	4,147	7,435	3.74	6.86×10^{-24}	1
	0.87-1.92	1,395	45,937	2.35	1.0×10^{-5}	2
	1.1	566	603	3.54	1.0×10^{-4}	3
	1.25	1,856	4,955	3.14	6.48×10^{-11}	4
	1.3	4,572	3,286	2.53	8.64×10^{-38}	5
All brain tumors	1.92	327	20,824	2.18	1.8×10^{-3}	2
Neuroblastoma	0.1-1.3	2,801	7,446	2.3	2.03×10^{-11}	6
Esophageal squamous cell carcinoma	1.0	405	810	3.22	1.34×10^{-4}	7
Skin basal cell carcinoma	0.46-1.92	4,319	51,810	2.16	2.2×10^{-20}	2
Prostate cancer	0.28-1.92	7,790	50,629	1.44	2.4×10^{-6}	2
Colorectal adenoma	1.85-1.92	4,095	43,022	1.39	1.6×10^{-4}	2
Breast cancer	0.5-1.91	4,985	42,995	1.06	0.57	2
High-risk breast cancer[3]	0.5-1.91	1,739	40,250	0.87	0.36	2
Melanoma	0.5-1.91	2,520	46,644	1.07	0.64	2
	1.39	1,329	3,000	1.15	0.680	8
Lung cancer	1.04	1,013	3,000	0.84	0.379	8
Squamous cell carcinoma of head and neck	0.59	1,096	3,000	0.44	0.008	8
Uterine leiomyoma	1.8	16,595	523,330	1.74	4.0×10^{-37}	9
All cancers (meta-analysis)		36,599	91,272	1.51	<0.001	10

[1] MAF, minor allele frequency in the control groups.
[2] OR, odds ratio.
[3] High-risk breast cancer is defined as breast cancer diagnosed before age of 50 or that having a history of multiple primary breast tumors.

Supplementary Table 2. Summary of B-cell malignancies developed in mutant mice.

B cell malignancy type	Number of animals (%)	
	p53$^{+/C}$	***p53***$^{C/C}$
Follicular lymphoma	13 (37)	11 (31)
Diffuse large B-cell lymphoma	7 (20)	6 (17)
Splenic marginal zone lymphoma	2 (5.7)	5 (14)
Plasmacytoma	2 (5.7)	1 (2.8)
Malignant plasmacytoma	0	1 (2.8)

Supplementary Table 3. *p53* coding mutations in tumors developed in *p53*$^{+/C}$ and *p53*$^{C/C}$ mice

Mouse ID[1]	Genotype	Disease[2]	Non-synonymous mutations[3]	Ortholog in human *TP53*
1	*p53*$^{+/C}$	FL	P82T	P85T
2	*p53*$^{+/C}$	FL	S212R	S215R
3	*p53*$^{+/C}$	FL	Y199C	R202C
4	*p53*$^{+/C}$	DLBCL	G182S	S185S
5	*p53*$^{+/C}$	SMZL	L46M	L45M
6	*p53*$^{C/C}$	SMZL	Q70K	V73K
7	*p53*$^{C/C}$	DLBCL	Q19. ∗	Q16. ∗
8	*p53*$^{C/C}$	FL	P47S	D47S
9	*p53*$^{C/C}$	DLBCL	E32G	N29G
10	*p53*$^{C/C}$	DLBCL	P38T	P46T
11	*p53*$^{C/C}$	FL	P38T	P46T
12	*p53*$^{C/C}$	SMZL	E55. ∗	E56. ∗
13	*p53*$^{C/C}$	SMZL	H230N	H233N

[1] Mouse IDs are the same as in Supplementary Fig. 1d.
[2] FL, follicular lymphoma; DLBCL, diffuse large B-cell lymphoma; SMZL, splenic marginal zone lymphoma.
[3] Major *p53* coding mutants identified by exome sequencing. ∗, stop codon.

Supplementary Table 4. Mouse line crosses and genotypes selected

Parent genotype (Genetic background)	Offspring genotype	Offspring genetic background	Offspring Selected for study
$p53^{+/C}$ × $p53^{+/C}$ (C57 × C57)	$p53^{+/+}$	C57	Yes
	$p53^{+/C}$	C57	Yes
	$p53^{C/C}$	C57	Yes
$p53^{+/–}$ × $p53^{+/–}$ (C57 × C57)	$p53^{+/+}$	C57	Yes
	$p53^{+/–}$	C57	Yes
	$p53^{–/–}$	C57	Yes
$p53^{+/C}$ × *PyVT* * *(F1)* (C57 × FVB)	$p53^{+/+}$;*PyVT*	50% FVB +50% C57	Yes
	$p53^{+/C}$;*PyVT*	50% FVB +50% C57	Yes
	$p53^{+/+}$	50% FVB +50% C57	No
	$p53^{+/C}$	50% FVB +50% C57	No
$p53^{+/C}$ × $p53^{+/C}$;*PyVT* * *(F2)* (C57 × [50% FVB +50% C57])	$p53^{+/+}$;*PyVT*	25% FVB +75% C57	Yes
	$p53^{+/C}$;*PyVT*	25% FVB +75% C57	Yes
	$p53^{C/C}$;*PyVT*	25% FVB +75% C57	Yes
	$p53^{+/+}$	25% FVB +75% C57	No
	$p53^{+/C}$	25% FVB +75% C57	No
	$p53^{C/C}$	25% FVB +75% C57	No
$p53^{+/C}$ × *Erbb2* * (C57 × FVB)	$p53^{+/+}$; *Erbb2*	50% FVB +50% C57	Yes
	$p53^{+/C}$; *Erbb2*	50% FVB +50% C57	Yes
	$p53^{+/+}$	50% FVB +50% C57	No
	$p53^{+/C}$	50% FVB +50% C57	No
$p53^{+/–}$ × *Erbb2* * (C57 × FVB)	$p53^{+/+}$; *Erbb2*	50% FVB +50% C57	Yes
	$p53^{+/–}$; *Erbb2*	50% FVB +50% C57	Yes
	$p53^{+/+}$	50% FVB +50% C57	No
	$p53^{+/–}$	50% FVB +50% C57	No

* Offspring of these crosses may or may not carry the indicated transgene (*PyVT* or *Erbb2*). C57, C57BL/6.

References

1. Enciso-Mora, V. *et al.* Low penetrance susceptibility to glioma is caused by the TP53 variant rs78378222. *Br J Cancer* **108**, 2178-2185 (2013).
2. Stacey, S.N. *et al.* A germline variant in the TP53 polyadenylation signal confers cancer susceptibility. *Nat Genet* **43**, 1098-1103 (2011).
3. Egan, K.M. *et al.* Rare TP53 genetic variant associated with glioma risk and outcome. *J Med Genet* **49**, 420-421 (2012).
4. Wang, Z. *et al.* Further confirmation of germline glioma risk variant rs78378222 in TP53 and its implication in tumor tissues via integrative analysis of TCGA data. *Hum Mutat* **36**, 684-688 (2015).
5. Melin, B.S. *et al.* Genome-wide association study of glioma subtypes identifies specific differences in genetic susceptibility to glioblastoma and non-glioblastoma tumors. *Nat Genet* **49**, 789-794 (2017).
6. Diskin, S.J. *et al.* Rare Variants in TP53 and Susceptibility to Neuroblastoma. *J Natl Cancer Ins* **106**, dju047 (2014).
7. Zhou, L., Yuan, Q. & Yang, M. A functional germline variant in the P53 polyadenylation signal and risk of esophageal squamous cell carcinoma. *Gene* **506**, 295-297 (2012).
8. Guan, X., Wang, L.-E., Liu, Z., Sturgis, E.M. & Wei, Q. Association between a rare novel TP53 variant (rs78378222) and melanoma, squamous cell carcinoma of head and neck and lung cancer susceptibility in non-Hispanic Whites. *J Cell Mol Med* **17**, 873-878 (2013).
9. Rafnar, T. *et al.* Variants associating with uterine leiomyoma highlight genetic background shared by various cancers and hormone-related traits. *Nat Commun* **9**, 3636 (2018).
10. Wang, Y. *et al.* A novel TP53 variant (rs78378222 A > C) in the polyadenylation signal is associated with increased cancer susceptibility: evidence from a meta-analysis. *Oncotarget* **7**, 32854-32865 (2016).
