# Supplementary material for: Tissue-specific microRNA expression alters cancer susceptibility conferred by a TP53 noncoding variant
Source: Nat Commun. 2019 Nov 7;10:5061. doi: 10.1038/s41467-019-13002-x (PMC6838078; doi:10.1038/s41467-019-13002-x)
Supplement: Supplementary file 2 — Reporting Summary [file 41467_2019_13002_MOESM2_ESM.pdf]

nature research

Corresponding author(s): Yong Li

Last updated by author(s): Oct 3, 2019

Reporting Summary

Nature Research wishes to improve the reproducibility of the work that we publish. This form provides structure for consistency and transparency in reporting. For further information on Nature Research policies, see Authors & Referees and the Editorial Policy Checklist.

Statistics

For all statistical analyses, confirm that the following items are present in the figure legend, table legend, main text, or Methods section.

n/a	Confirmed	
☐	☒	The exact sample size (*n*) for each experimental group/condition, given as a discrete number and unit of measurement
☐	☒	A statement on whether measurements were taken from distinct samples or whether the same sample was measured repeatedly
☐	☒	The statistical test(s) used AND whether they are one- or two-sided<br>*Only common tests should be described solely by name; describe more complex techniques in the Methods section.*
☐	☒	A description of all covariates tested
☒	☐	A description of any assumptions or corrections, such as tests of normality and adjustment for multiple comparisons
☐	☒	A full description of the statistical parameters including central tendency (e.g. means) or other basic estimates (e.g. regression coefficient) AND variation (e.g. standard deviation) or associated estimates of uncertainty (e.g. confidence intervals)
☒	☐	For null hypothesis testing, the test statistic (e.g. *F*, *t*, *r*) with confidence intervals, effect sizes, degrees of freedom and *P* value noted<br>*Give P values as exact values whenever suitable.*
☒	☐	For Bayesian analysis, information on the choice of priors and Markov chain Monte Carlo settings
☒	☐	For hierarchical and complex designs, identification of the appropriate level for tests and full reporting of outcomes
☒	☐	Estimates of effect sizes (e.g. Cohen's *d*, Pearson's *r*), indicating how they were calculated

Our web collection on statistics for biologists contains articles on many of the points above.

Software and code

Policy information about availability of computer code

Data collection	miRBase, miRGator, miRNAmap, miRmine, TargetScan, PolymiRTS
Data analysis	Prism GraphPad 5/7 and SigmaStat

For manuscripts utilizing custom algorithms or software that are central to the research but not yet described in published literature, software must be made available to editors/reviewers. We strongly encourage code deposition in a community repository (e.g. GitHub). See the Nature Research guidelines for submitting code & software for further information.

Data

Policy information about availability of data

All manuscripts must include a data availability statement. This statement should provide the following information, where applicable:

- Accession codes, unique identifiers, or web links for publicly available datasets
- A list of figures that have associated raw data
- A description of any restrictions on data availability

We have deposited data on p53 mutation in lymphoma in the NCBI BioProject database under the accession code PRJNA575686.

Field-specific reporting

Please select the one below that is the best fit for your research. If you are not sure, read the appropriate sections before making your selection.

☒ Life sciences ☐ Behavioural & social sciences ☐ Ecological, evolutionary & environmental sciences

For a reference copy of the document with all sections, see nature.com/documents/nr-reporting-summary-flat.pdf

Life sciences study design

All studies must disclose on these points even when the disclosure is negative.

Sample size	No statistical methods were used to predetermine sample size. The number of animals used in each experiment was estimated from published studies with statistically significant results.
Data exclusions	For survival studies, mice occasionally sacrificed for wounds caused by fighting and mice with other accidents with earlier death were excluded from analyses.
Replication	Three or more biological replications with three technical replications were performed for each in vitro study. For mammary tumorigenesis, two independent models were used.
Randomization	For tumor-free survival, mice were not randomized for studies; we used litter mates and mice were grouped by their genotype. When mice with the same genotype were subjected to different treatments, they were allocated into groups based on body weight.
Blinding	Investigators were blinded to group allocation during data collection.

Reporting for specific materials, systems and methods

We require information from authors about some types of materials, experimental systems and methods used in many studies. Here, indicate whether each material, system or method listed is relevant to your study. If you are not sure if a list item applies to your research, read the appropriate section before selecting a response.

Materials & experimental systems

n/a	Involved in the study	
☐	☒	Antibodies
☐	☒	Eukaryotic cell lines
☒	☐	Palaeontology
☐	☒	Animals and other organisms
☐	☒	Human research participants
☒	☐	Clinical data

Methods

n/a	Involved in the study	
☒	☐	ChIP-seq
☐	☒	Flow cytometry
☒	☐	MRI-based neuroimaging

Antibodies

Antibodies used	Antibodies against B220 (ab64100), Igκ light chain (ab190484), GFAP (ab7260), synaptophysin (ab32127), HA-tag (ab24779), PCNA (ab29), GD3 (ab11779), nestin (ab6142), olig2 (ab109186), E-cadherin (for IHC, ab76055), PCK (ab7753), p53 (for IHC, ab31333), phosphorylated-p53 Ser15 (for IHC, ab1431), vimentin (ab92547), NTP-II (ab33595) and α-SMA (ab5694) were purchased from Abcam (Cambridge, MA). Antibodies against p53 (for western blotting [WB], CST2524), phosphorylated-p53 Ser15 (for WB, CST9248), GAPDH (CST5174), and β-actin (CST4970) were from Cell Signaling Technology (Danvers, MA). Antibodies against β-casein (SC-166684) and β-catenin (SC-7963) were from Santa Cruz Biotechnology (Dallas, TX).
Validation	We generally validate each antibody and each lot of antibody for specificity. Validation includes western blotting with cell lines after transfection with specific siRNA and/or expression constructs. The number of immunoreactive bands and the size at which these bands migrate is determined. Whenever possible, results are compared with those obtained for another antibody raised against different epitopes of the investigated protein, or raised in a different species. Immunohistochemical and immunocytochemical staining is done to validate the cellular localization of the targeted protein. Positive and negative controls include tissues or cell lines that do not express the target protein, or have been shown before to (not) express the target genes. If warranted, specificity of antibody is verified also using mass spectrometry after immunoprecipitation.

Eukaryotic cell lines

Policy information about cell lines

Cell line source(s)	HCT116 (p53-/-) from Dr. Bert Vogelstein and all others from ATCC
Authentication	Cell line authentication is performed by ATCC using Short Tandem Repeat Profiling. p53-null in HCT116 (p53-/-) was confirmed by Western blotting.
Mycoplasma contamination	Yes. All cell lines were tested and were negative for mycoplasma.
Commonly misidentified lines (See ICLAC register)	No commonly misidentified cell lines maintained by ICLAC was used in this study.

Animals and other organisms

Policy information about studies involving animals; ARRIVE guidelines recommended for reporting animal research

Laboratory animals	We used both male and female mice in all experiments, except in mammary tumorigenesis studies where only females were used. When two or three groups were compared, we use F1 or F2 littermates from intercrossing.
Wild animals	None
Field-collected samples	None
Ethics oversight	All mouse studies were performed in compliance with procedures approved by the Institutional Animal Care and Use Committees at Cleveland Clinic and Wuhan Central Hospital.

Note that full information on the approval of the study protocol must also be provided in the manuscript.

Human research participants

Policy information about studies involving human research participants

Population characteristics	All recruited breast cancer and sarcoma patients, who are ethnic Han Chinese, were diagnosed at the Central Hospital of Wuhan, China.
Recruitment	Patients who were suspected to have LFS or hereditary breast were excluded. Controls were unaffected (not diagnosed with any cancer) and were recruited at the same hospital with age (in 10-year intervals), sex, and ethnicity matched to the cancer cases.
Ethics oversight	This study was approved by the Ethical and Scientific Committee of the Central Hospital of Wuhan, and all human subject research was performed in accordance with institutional, national, and Declaration of Helsinki requirements.

Note that full information on the approval of the study protocol must also be provided in the manuscript.

Flow Cytometry

Plots

Confirm that:

- [x] The axis labels state the marker and fluorochrome used (e.g. CD4-FITC).
- [x] The axis scales are clearly visible. Include numbers along axes only for bottom left plot of group (a 'group' is an analysis of identical markers).
- [x] All plots are contour plots with outliers or pseudocolor plots.
- [x] A numerical value for number of cells or percentage (with statistics) is provided.

Methodology

Sample preparation	Human cancer cell lines from ATCC were transfered with plasmids expressing EGFP/RFP carrying WT TP53 3’-UTR or mutant TP53 3’-UTR by using lipofectamine 3000. 48 hours later, single cell suspension was prepared.
Instrument	MACSQuant® Analyzer 10
Software	MACSQuantify software
Cell population abundance	>95%
Gating strategy	The preliminary FSC/SSC gates for starting cell population was determined by running cells transfered with control plasmids (without EGFP/RFP expression). The boundary of positive and negative cells was determine by comparing the fluorescence intensity of the same cells transferred with control plasmids or same amount of EGFP/RFP-expressing plasmids at channel 510/588 with the same instrument settings.

- [ ] Tick this box to confirm that a figure exemplifying the gating strategy is provided in the Supplementary Information.
