# Supplementary material for: Tissue-specific microRNA expression alters cancer susceptibility conferred by a TP53 noncoding variant
Source: Nat Commun. 2019 Nov 7;10:5061. doi: 10.1038/s41467-019-13002-x (PMC6838078; doi:10.1038/s41467-019-13002-x)

Source Data

Figure 3a (part 1)

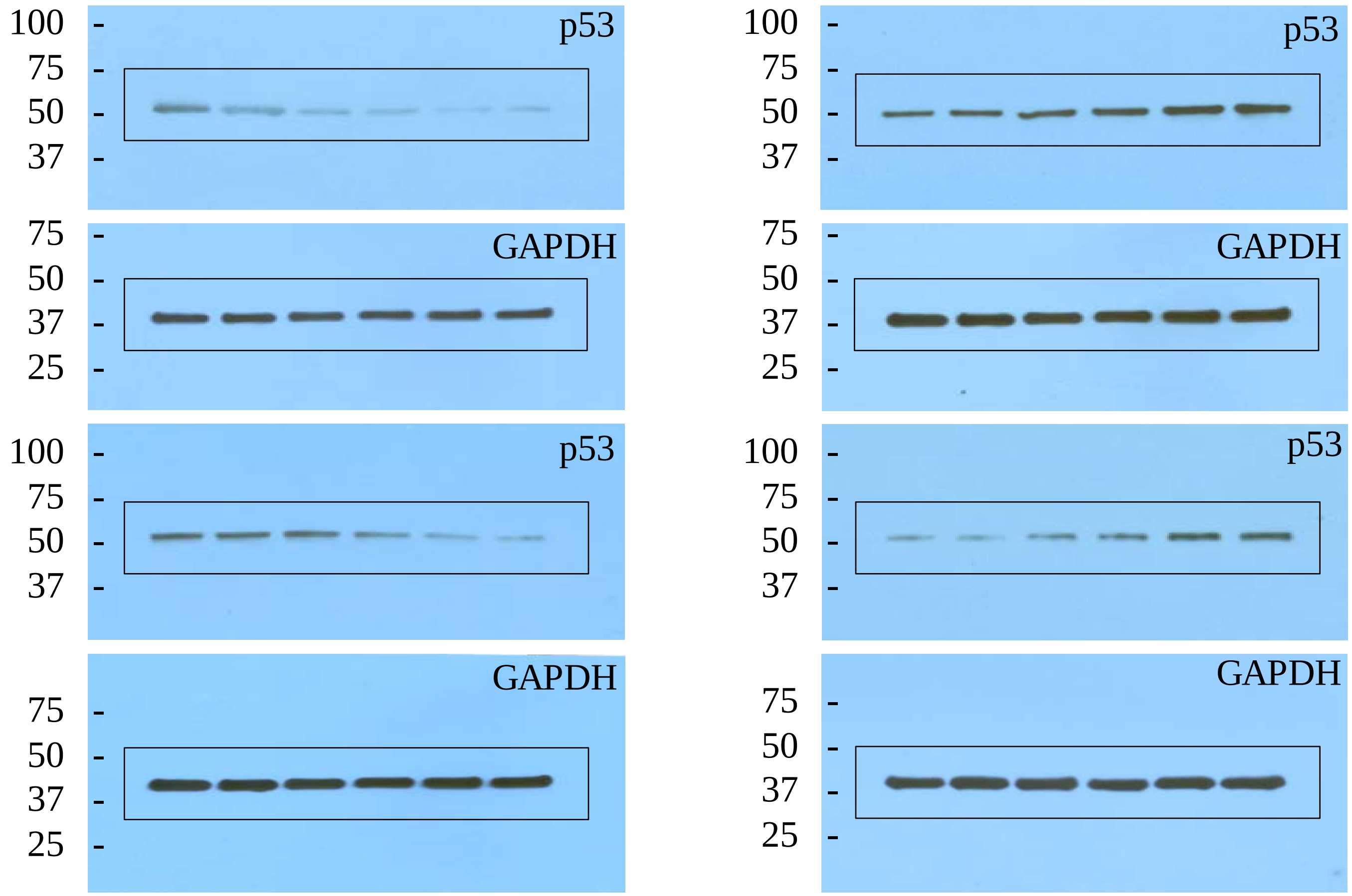

Figure 3a (part 2)

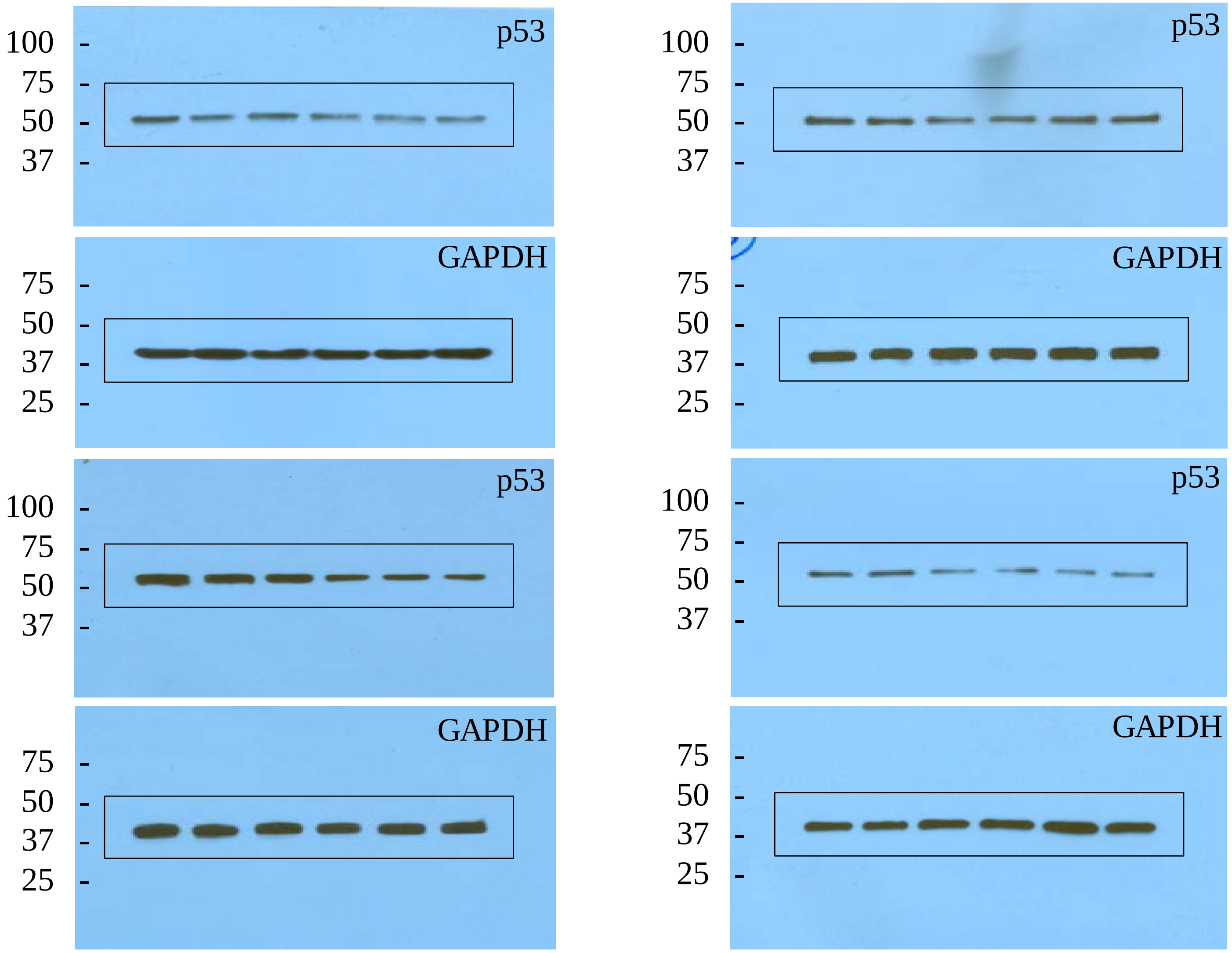

Figure 3f

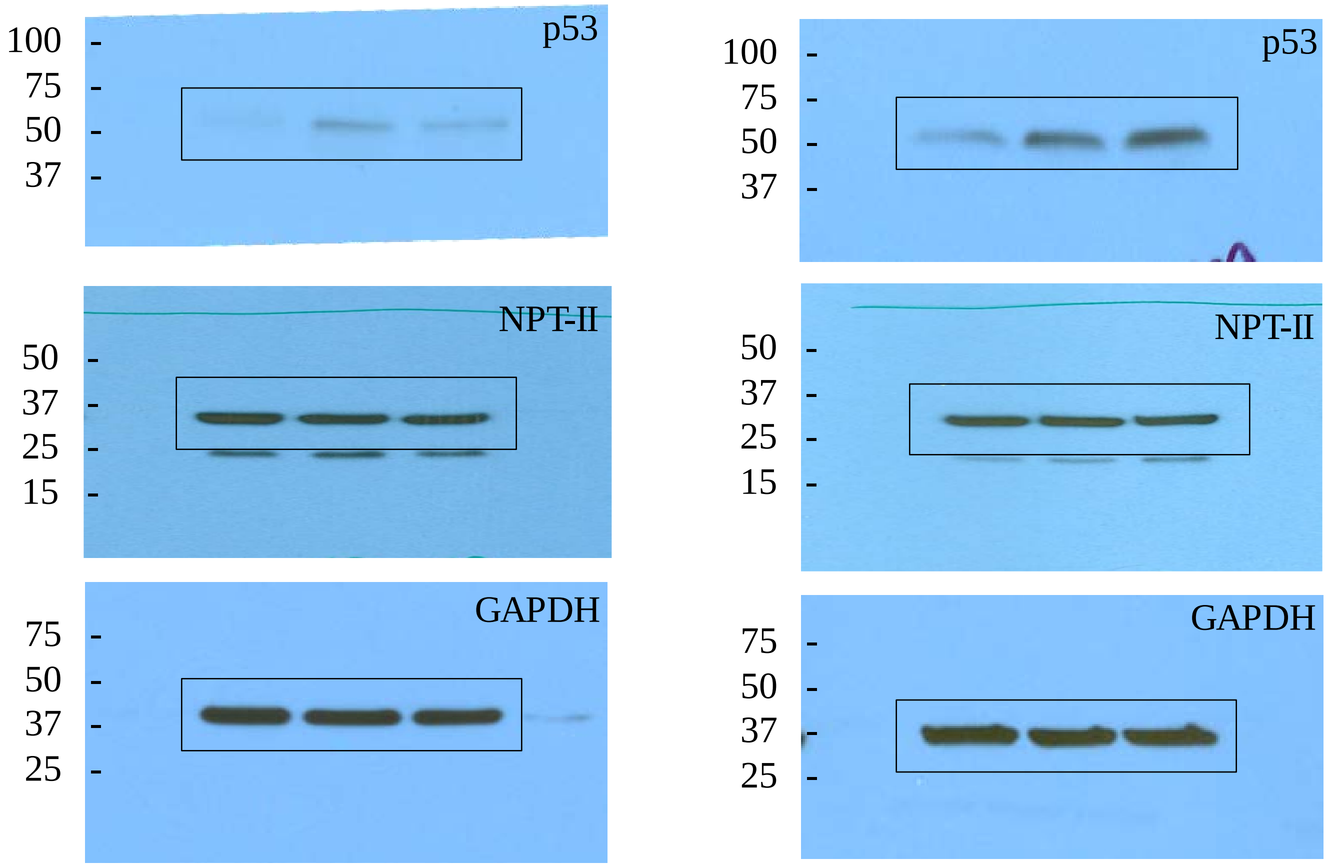
100
75
50
37
p53
50
37
25
15
NPT-II
75
50
37
25
GAPDH
100
75
50
37
p53
50
37
25
15
NPT-II
75
50
37
25
GAPDH

Figure 4e

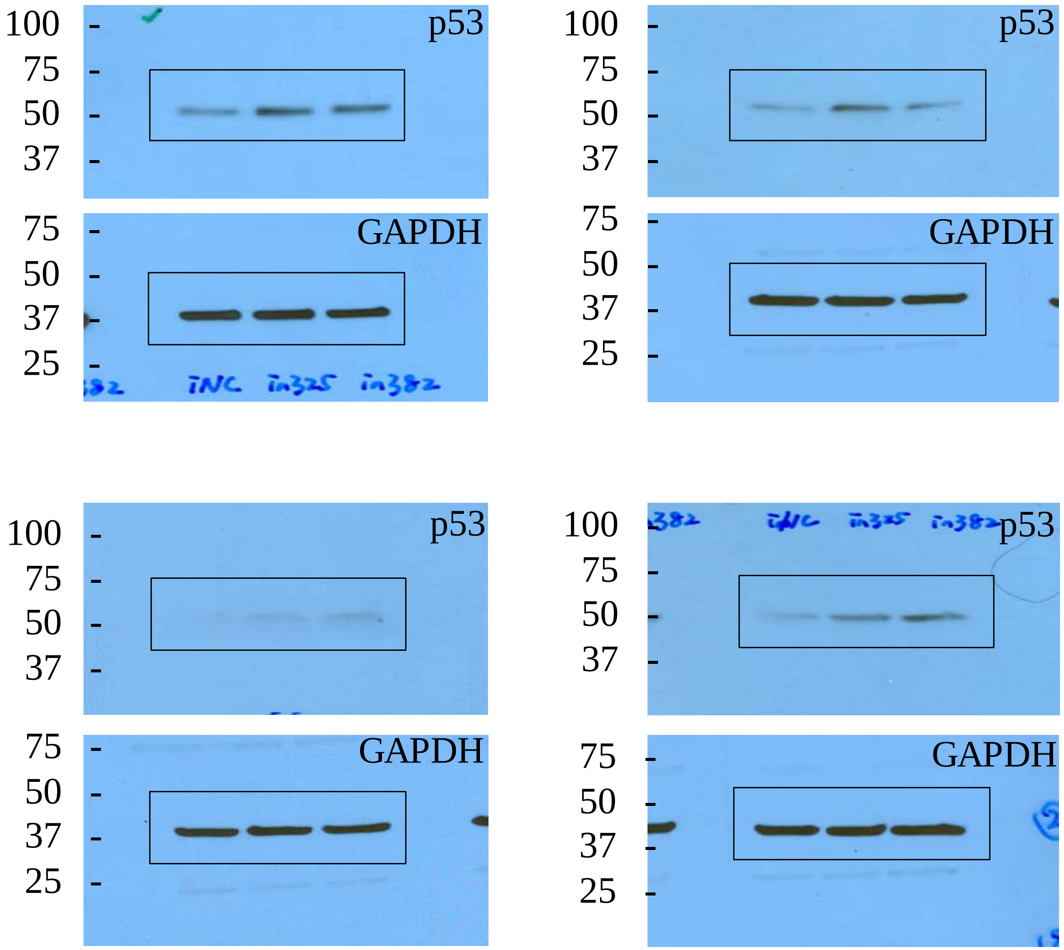

100
75
50
37
p53
75
50
37
25
GAPDH
100
75
50
37
p53
75
50
37
25
GAPDH
100
75
50
37
p53
75
50
37
25
GAPDH
100
75
50
37
p53
75
50
37
25
GAPDH

Figure 4f

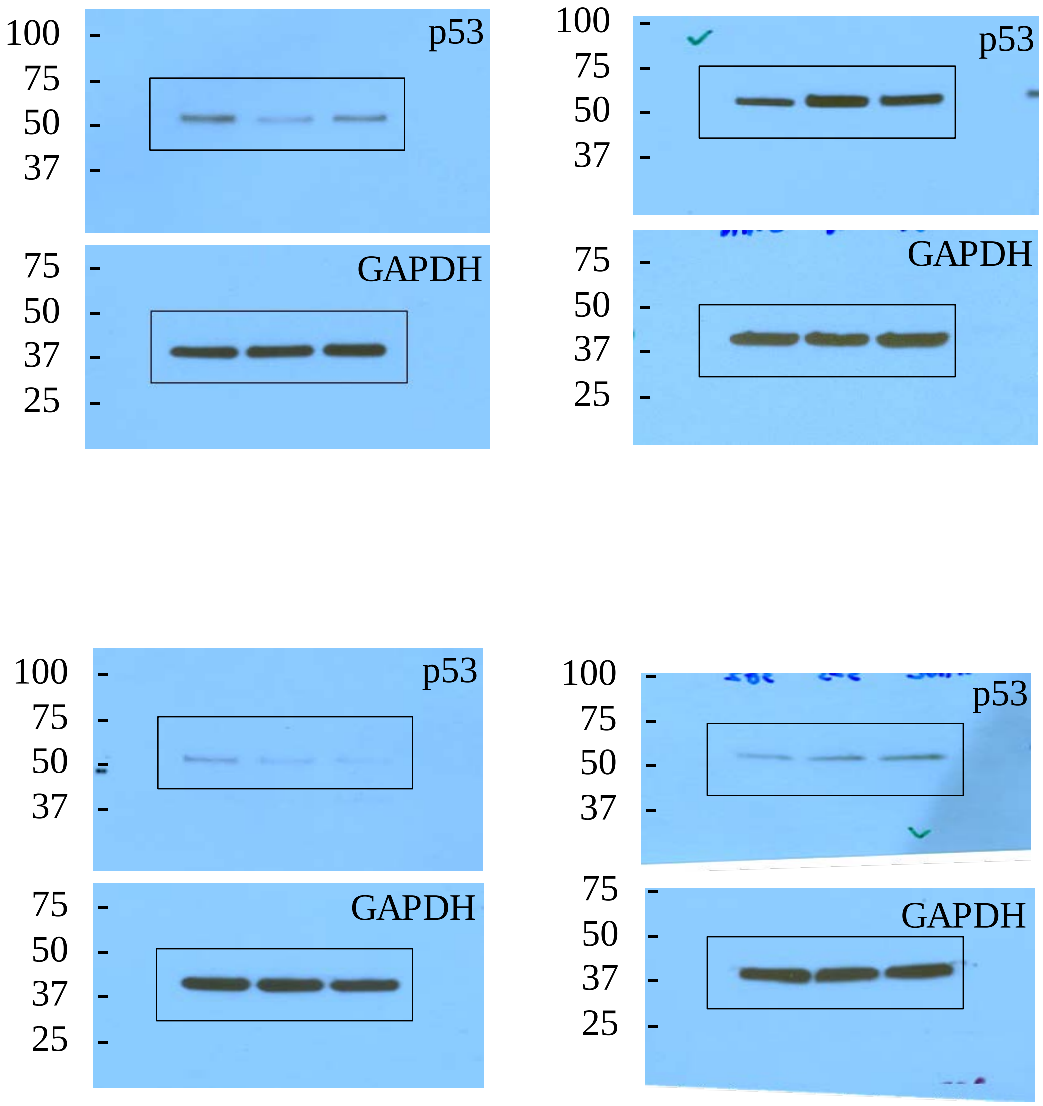
100
75
50
37
p53
75
50
37
25
GAPDH
100
75
50
37
p53
75
50
37
25
GAPDH
100
75
50
37
p53
75
50
37
25
GAPDH
100
75
50
37
p53
75
50
37
25
GAPDH

Supplemental Figure 6a, 6b

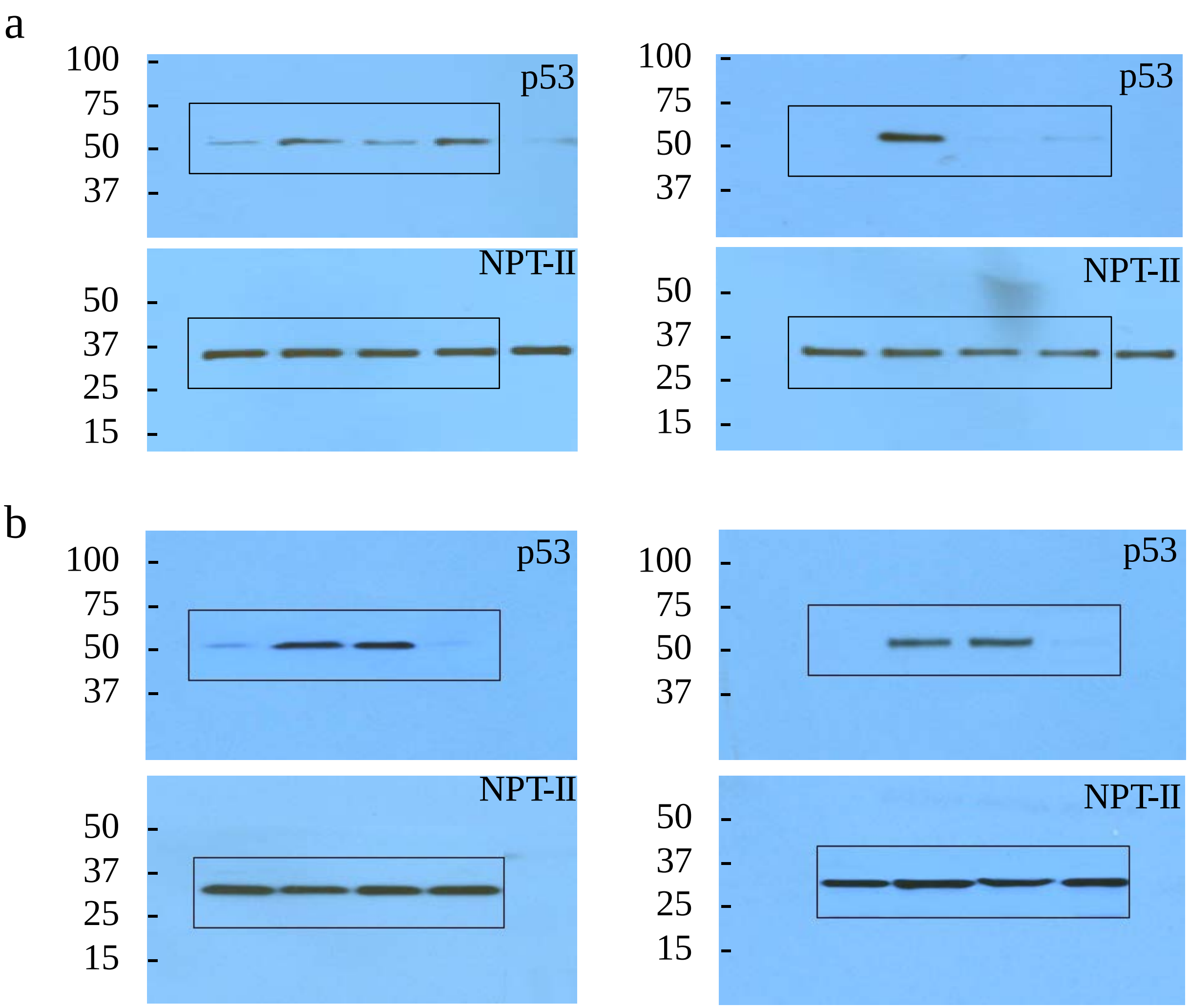

Supplemental Figure 6c, 6d

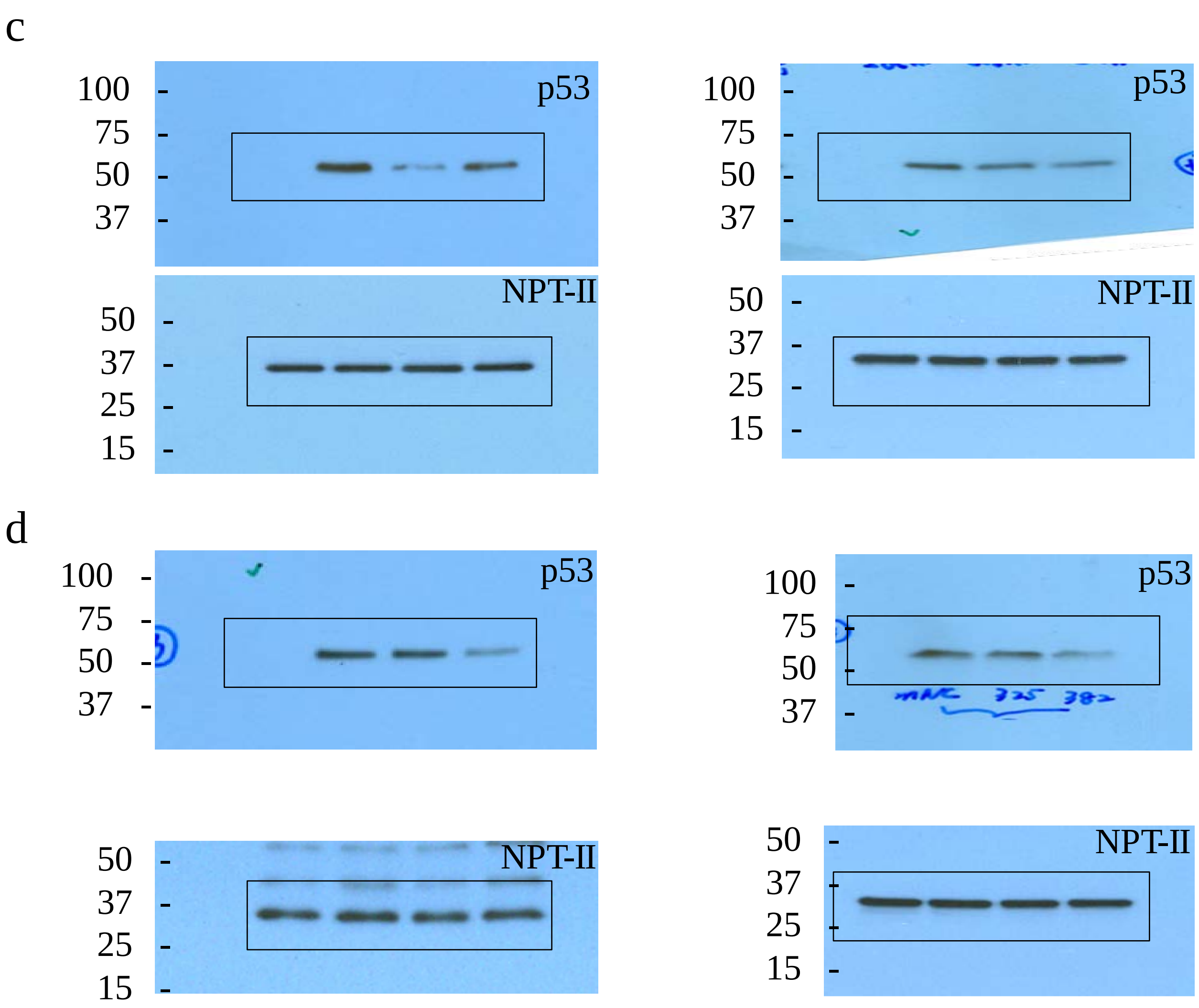

Supplement: Supplementary file 3 — Source Data [file 41467_2019_13002_MOESM3_ESM.pdf]
